# Highly Accurate and Efficient Deep Learning Paradigm for Full-Atom Protein Loop Modeling with KarmaLoop

**DOI:** 10.34133/research.0408

**Published:** 2024-07-25

**Authors:** Tianyue Wang, Xujun Zhang, Odin Zhang, Guangyong Chen, Peichen Pan, Ercheng Wang, Jike Wang, Jialu Wu, Donghao Zhou, Langcheng Wang, Ruofan Jin, Shicheng Chen, Chao Shen, Yu Kang, Chang-Yu Hsieh, Tingjun Hou

**Affiliations:** ^1^ Innovation Institute for Artificial Intelligence in Medicine of Zhejiang University, College of Pharmaceutical Sciences, Zhejiang University, Hangzhou 310058, Zhejiang, China.; ^2^ Zhejiang Laboratory, Hangzhou 311100, Zhejiang, China.; ^3^ Shenzhen Institute of Advanced Technology, Chinese Academy of Sciences, Shenzhen 518055, Guangdong, China.; ^4^ Department of Pathology, New York University Medical Center, New York, NY 10016, USA.; ^5^ College of Life Sciences, Zhejiang University, Hangzhou 310058, Zhejiang, China.

## Abstract

Protein loop modeling is a challenging yet highly nontrivial task in protein structure prediction. Despite recent progress, existing methods including knowledge-based, ab initio, hybrid, and deep learning (DL) methods fall substantially short of either atomic accuracy or computational efficiency. To overcome these limitations, we present KarmaLoop, a novel paradigm that distinguishes itself as the first DL method centered on full-atom (encompassing both backbone and side-chain heavy atoms) protein loop modeling. Our results demonstrate that KarmaLoop considerably outperforms conventional and DL-based methods of loop modeling in terms of both accuracy and efficiency, with the average RMSDs of 1.77 and 1.95 Å for the CASP13+14 and CASP15 benchmark datasets, respectively, and manifests at least 2 orders of magnitude speedup in general compared with other methods. Consequently, our comprehensive evaluations indicate that KarmaLoop provides a state-of-the-art DL solution for protein loop modeling, with the potential to hasten the advancement of protein engineering, antibody–antigen recognition, and drug design.

## Introduction

Loops are irregular segments in protein structures that often link 2 regular secondary structures such as α helices or β sheets [1,2]. These structures are typically situated near the surface of proteins [3], positioned in a way to allow them to facilitate numerous essential biological functions [4], including regulation of enzyme activity [5–8], protein–protein interaction (PPI) [9], and protein-ligand recognition [10–12]. Given their high flexibility, loops heavily influence the overall protein dynamics [13], rendering loop prediction one of the most challenging yet indispensable tasks in protein modeling [14]. Furthermore, since over half of the experimentally determined structures in the Research Collaboratory for Structural Bioinformatics Protein Data Bank (RCSB PDB) [15] contain missing fragments, and these missing regions predominantly correspond to loops [4], accurate modeling of loop structures is of high importance. For example, the prediction of antibody complementarity determining region (CDR) H3 loops is the most challenging task in antibody modeling [16], mainly due to the much higher flexibility of H3 compared with other CDR loops. To address this issue, various computational methods including traditional methods (knowledge-based, ab initio, and hybrid) and deep learning (DL) methods have been proposed. Knowledge-based methods, such as FREAD [17], LoopIng [18], SuperLooper [19], and DaReUS-Loop [20], rely on template repositories collected from pre-existing protein structures and perform modeling based on certain standards, such as structural similarity. For instance, FREAD performs database searching using the 4 rule-based filters based on anchor C_α_ separations and the environmentally constrained substitution score. LoopIng selects templates from the database by taking advantage of both sequence- and geometry-related features. SuperLooper provides the first online server for modeling globular and membrane protein loops, where the loop candidates are chosen by the standard of the root mean square deviation (RMSD) of the stem atoms. DaReUS-Loop uses fragments from remote or unrelated proteins to complete loop modeling and filters by sequence similarity and conformational profile. These methods are widely used in loop modeling, particularly in cases where a comparable fragment is available in the template database. However, the modeling performance of these methods for novel structures (especially for long loops) would drop sharply without proper references, suggesting that the accuracy of these methods is severely limited by the available templates [21,22].

Ab initio methods, mainly consisting of 2 components, conformational sampling and scoring, including DISGRO [1], LEAP [23], cyclic coordinate descent (CCD) [24,25], robotics-based kinematic closure (KIC) [26], and next-generation KIC (NGK) [27], offer advantages over knowledge-based methods due to their independence from templates and the ability to explore a broader conformational space. However, they need more computational resources than knowledge-based methods, and their computational cost increases exponentially with the loop length [21]. DISGRO, for instance, employs a chain growth sequential Monte Carlo sampling strategy for loop modeling and an atom-based distance-dependent empirical potential scoring function specifically designed for loops, and it has been described as an efficient computational method and particularly effective for modeling loops with 10 to 17 residues. CCD minimizes the distances between the backbone atoms of the C-terminal moving anchor and the corresponding atoms in the fixed anchor by adjusting one dihedral angle at a time. KIC utilizes a robotics-inspired local loop reconstruction method for loop modeling, achieving sub-angstrom accuracy. Among ab initio methods, NGK has been commonly regarded to be the state of the art [20,21], as it combines intensification and annealing strategies and yields a 4-fold improvement over standard KIC.

Hybrid methods, such as CODA [28] and Sphinx [22], integrate both ab initio and knowledge-based methods to improve performance [20]. CODA utilizes a combination of the knowledge-based approach FREAD and the ab initio method PETRA [29] for generating a consensus prediction that should satisfy a set of rule-based filters. Sphinx combines FREAD and an in-house ab initio method that first selects potential fragments shorter than the length of loop to be modeled. Then, the ab initio method adjusts the length of the fragments to generate accurate decoys of the desired length.

With the continual development of computing resources, DL has become increasingly popular and has demonstrated its potential in predicting protein structures. For instance, AlphaFold [30] incorporates biological and physical information and utilizes multi-sequence alignments (MSAs) to design a DL algorithm, achieves atomic-level accuracy, and ranks first in the 14th Critical Assessment of Structure Prediction (CASP) competition. Likewise, RoseTTAFold [31] utilizes a 3-track neural network that simultaneously considers protein sequences, amino acid interactions, and structures to attain accuracy close to AlphaFold [32]. However, both methods often face great challenges in accurately modeling loop regions [32,33], which is related to the fact that these methods typically do not incorporate factors such as ligand presence, the complexities of PPIs, and multiple conformations [34]. AlphaFold-Multimer [35] is developed as a useful tool in protein complex prediction and exhibits outstanding performance in antibody structure prediction [36]. More recently, AlphaFold3 [37] has shown remarkable capabilities in extending more accurate structural predictions to a broader scope that includes interactions with ligands, proteins, nucleic acids, and posttranslational modifications. Despite the need for further validation from the scientific community, these advancements in AlphaFold3 have brought great hope to this field.

The first DL method particularly for loop modeling was proposed in 2019 [38], which employs convolutional neural networks (CNNs) [39] on the distance matrix to predict the pairwise distances of loop atoms for conformation reconstruction. Recently, 2 DL architectures for loop modeling have been proposed [40], including one using a combined CNN–recursive neural network (RNN) structure (DeepMUSICS) and the other based on a refinement of histograms using a two-dimensional (2D) CNN architecture (DeepHisto). However, the source code of the aforementioned methods is not publicly available, which limits their broader applications. In the case of CDR H3 loop modeling, DeepH3 [41] was introduced using CNN to predict the interresidue distances and orientations from antibody sequences. Furthermore, DeepAb [42], the updated version of DeepH3, has demonstrated improvements with the inclusion of a pre-trained antibody sequence model and an interpretable attention mechanism to accurately predict the structures of antibodies.

So far, both traditional and DL-based methods have been unable to achieve high accuracy and efficiency simultaneously in loop prediction. Moreover, most proposed methods have focused solely on the prediction accuracy of heavy atoms in the backbone within the loop region [1,24,43–47]. Nonetheless, it is crucial and challenging to predict the conformation of side chains accurately [48–50], which highlights the importance of executing meticulous full-atom modeling of loops.

**Fig. 1.**
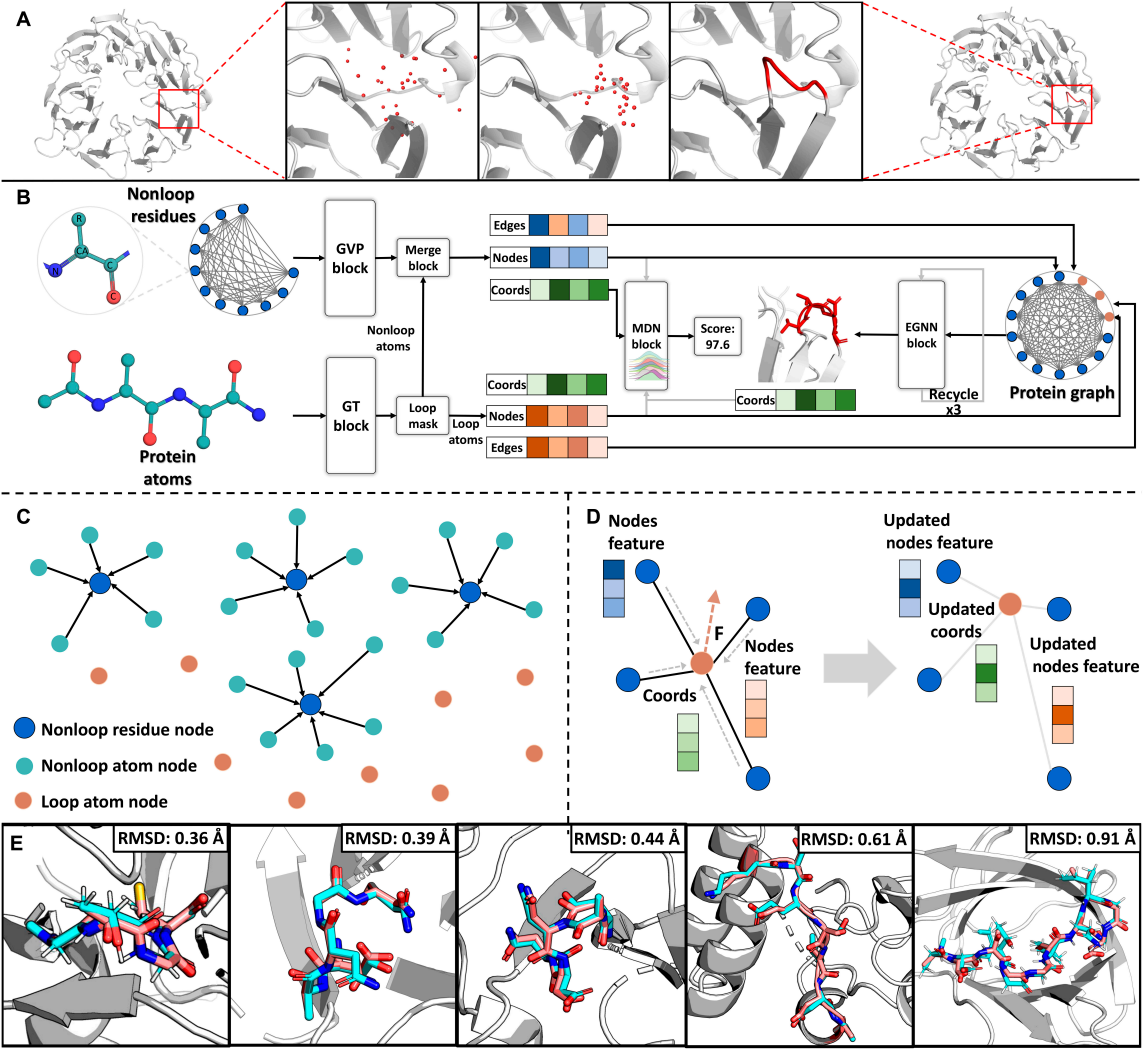
The workflow of KarmaLoop for loop modeling. (A) A loop modeling example, from a protein having a missing loop region to a whole protein with the predicted loop coordinates. (B) KarmaLoop architecture: the GT block consists of multi-head attention layers of the GT, while the GVP block represents GVP. The merge block indicates the layers of merging the nonloop atom node features into the corresponding residue node features, and the Loop Mask denotes the process of distinguishing loop nodes from nonloop nodes. The architecture incorporates the MDN denoted as the MDN block and the E(n) EGNN layer denoted as the EGNN block. (C) Process of merging nonloop atom node features and residue node features. The nonloop residue node is represented by a dark blue dot with a black frame, the nonloop atom node feature is represented by a light blue dot, and the loop atom node feature is represented by a tawny dot. (D) Message-passing scheme of EGNN: the node features. The graph on the left is the initial state of each node, and the graph on the right is the updated state. During the message-passing process, both node features, edge features, the node distances, and the node directions form the messages passed along edges and the pseudo-force F for coordinate updating, resulting in the features and coordinates of the updated nodes. (E) The structures of 5 KarmaLoop-generated loops are displayed, with the pink representing the KarmaLoop-predicted conformation and the blue indicating the ground truth (CASP IDs and loop lengths from left to right: T1129s2-D1: 4 amino acids, T1145-D2: 4 amino acids, T1056: 4aa, T1044: 7 amino acids, and T1137s3-D1: 10 amino acids). All samples are taken from CASP13, CASP14, and CASP15.

For the reasons above, we present a novel end-to-end DL paradigm named KarmaLoop, which is designed for accurate and efficient full-atom prediction of protein loops. Figure 1A depicts an example of a loop modeled by KarmaLoop, which starts with a random initialization of the loop coordinates and then predicts an accurate loop structure. KarmaLoop (Fig. 1B) employs graph neural network (GNN) architectures, which comprise 2 encoders [i.e., Graph Transformer (GT) [51–53] and Geometric Vector Perceptrons (GVPs) [54]] for intramolecular interaction encoding, a mixture density network (MDN) block is employed to assess confidence levels in the predictions, and an E(n) equivariant GNN (EGNN) block is used to generate the loop conformation. While MDN is adept at distinguishing between high-RMSD and low-RMSD conformations, it does not display a satisfactory linear correlation with the RMSD results. The innovations are as follows: (a) better representation: by integrating atom features within their respective residues, we achieve a more holistic representation of proteins (Fig. 1C); (b) enhanced accuracy: the MDN block is trained prior to the introduction of the EGNN to better learn the distribution of distances between loop atoms and nonloop residues, thereby refining the learning process for pose generation; (c) higher efficiency: instead of relying on searching algorithms, loop conformations are updated using pseudo-forces as predicted by EGNN, thereby accelerating the modeling process.

KarmaLoop has been evaluated on multiple benchmark datasets, including the general protein loop benchmark CASP13+14 (the combination of CASP13 [55] and CASP14 [56]), the CASP15 dataset, and the antibody H3 loop benchmark (RosettaAntibody and therapeutic benchmark used in DeepAb), and filter the samples with high sequence similarity with the training dataset. In terms of the general protein loop modeling capability, KarmaLoop outperforms all the tested traditional and DL-based methods on both accuracy and efficiency, with the medium full-atom RMSDs of 1.41 and 1.31 Å, the average RMSDs of 1.77 and 1.95 Å, the success rates of 75.09% and 71.71% for single-conformation generation and 77.51% and 76.21% for generating 5 conformations, and the speeds of 0.047 s and 0.049 s per task on the CASP13+14 and CASP15 benchmark datasets, respectively. Several successful predictions of antibody CDR H3 loops are shown in Fig. 1E, where KarmaLoop achieves the average full-atom and medium RMSDs of 3.25 and 2.97 Å, respectively, compared with the baseline methods with the best performance (average and medium RMSDs of 3.49 and 3.34 Å, respectively). In protein engineering and structure-based drug design, accurately modeling protein loops under an imprecise structural environment holds tremendous potential, but precisely reconstructing loop structures within an accurate framework structure is also quite important, a focus that has been extensively explored in most previous studies. To provide a comprehensive assessment, we also conducted the task of antibody H3 modeling based on inaccurate structural frameworks predicted by other methods. When using the AlphaFold-Multimer, AlphaFold, and DeepAb modeled antibody structures as the input, KarmaLoop could further refine the conformations of H3 loops and increase the accuracy by an average of 9%. Hence, we proposed a universal protocol for precise antibody modeling by employing DeepAb to first predict an overall antibody structure, followed by KarmaLoop to refine the H3 loops. In summary, KarmaLoop has shown a remarkable superiority (over prior arts) in predicting both general loops and antibody H3 loops across most loop lengths.

## Results and Discussion

As shown in Fig. 1B, KarmaLoop consists of 3 components: (a) 2 encoders, designed to comprehend representations of both residues and atoms, thereby implementing a hierarchical representation; (b) E(n) EGNNs enhanced with self-attention, aiming at updating the loop conformation by considering both intermolecular and intramolecular interactions; (c) an MDN designed for confidence evaluation and introducing the prior knowledge to facilitate prediction. To assess the ability of KarmaLoop, it is critical to make comparisons with other widely used methods in terms of prediction accuracy and efficiency. Consequently, in the subsequent sections, we conducted some experiments to evaluate the performance including the accuracy of generating conformations, the effect of loop length on accuracy, computational efficiency, the capacity for antibody H3 loop modeling, and 2 cases to prove that KarmaLoop is capable of reproducing diverse conformations according to varying ligand-binding conditions.

### Prediction accuracy for general protein loops

The accuracy of loop modeling is of utmost importance for many practical applications. To validate the capability of our model, 7 widely used methods including 3 DL-based methods (AlphaFold, RoseTTAFold, and ColabFold [57], and we used a customized template database that contains all the nonloop structures in the CASP13+14 and CASP15 benchmarks), one knowledge-based method (FREAD), and 3 ab initio methods [DISGRO, NGK, and Rosetta model_missing_loop (RML) [58]] were employed for comparison with KarmaLoop in a comprehensive benchmark. The full-atom RMSDs were calculated for each method, with the AlphaFold, ColabFold, and RoseTTAFold predicted structures being aligned before computation.

**Fig. 2.**
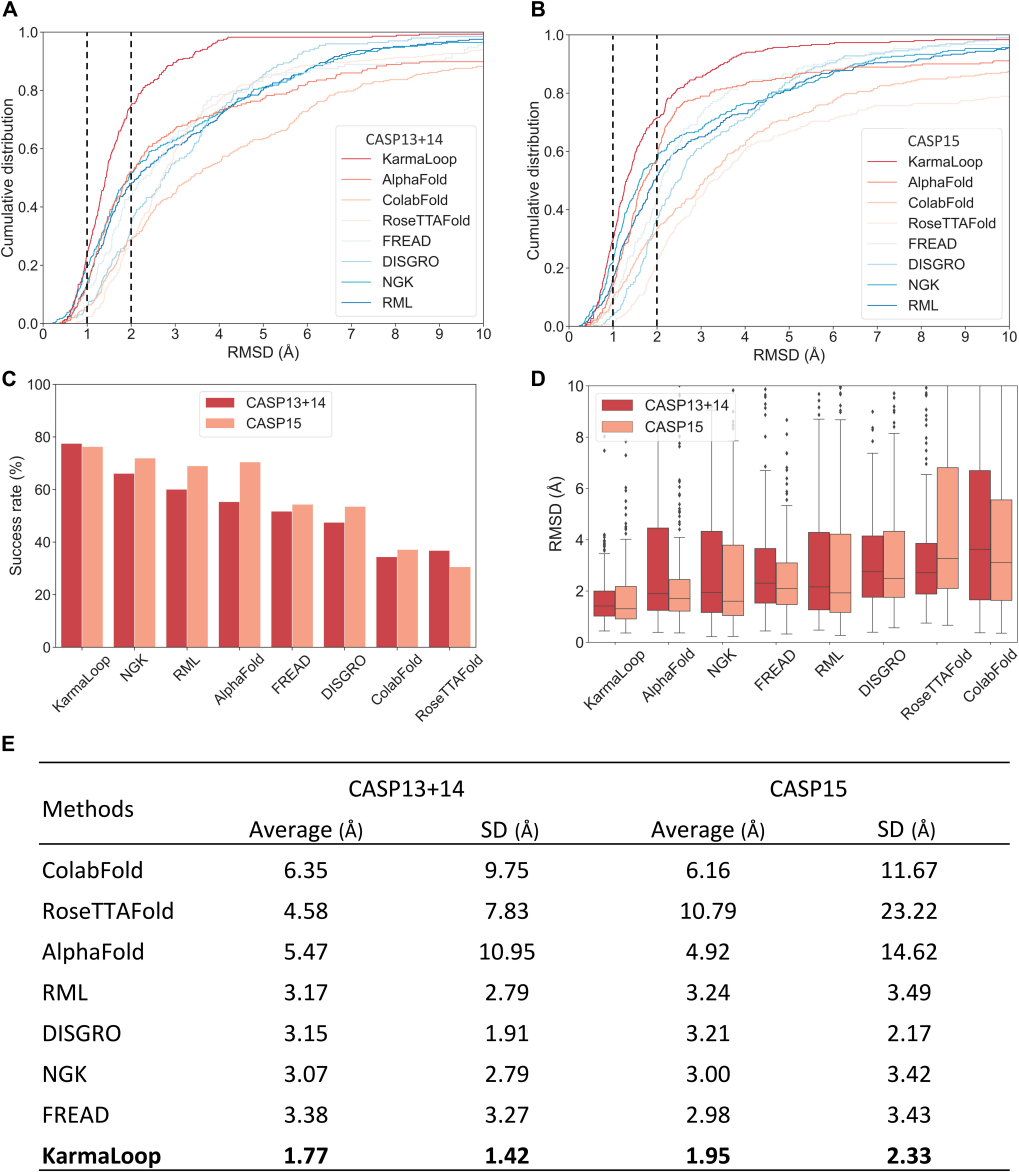
Performance of KarmaLoop and protein loop modeling methods on the CASP dataset. (A) Empirical cumulative distribution of RMSD of the tested methods on (A) the CASP13+14 dataset and (B) the CASP15 dataset, and the dashed lines indicate the 1- and 2-Å RMSD cutoff. (C) The success rate was calculated in the 2-Å RMSD threshold, with all the methods generating 5 conformations. (D) RMSD distribution of the tested methods. The red boxes indicate the CASP13+14 dataset, and the salmon boxes indicate the CASP15 dataset. (E) Average and SD of the RMSDs of all the tested methods.

To perform quantitative comparisons with the other methods, empirical cumulative distribution function (eCDF) was selected to describe the proportion of samples at given RMSD values. We defined a task as successful if the RMSD of a given sample falls below a specified threshold. Figure 2A and B demonstrates that when the RMSD threshold was set to 2 Å, KarmaLoop exhibited the highest success rates of 75.09% and 71.71%, respectively, on the CASP13+14 and CASP15 benchmark datasets, yielding impressive improvements by 22.92% and 14.30% over the next best method (i.e., AlphaFold, 52.17%; NGK, 57.41%). Even adopting a stricter RMSD standard of 1 Å, KarmaLoop (23.87% and 29.93%) still outperformed the other methods, ranging from RoseTTAFold (1.74% and 1.07%) to NGK (19.23% and 22.26%).

As some baseline methods may generate multiple conformations for a given loop sample, KarmaLoop was further evaluated in the context of generating multiple conformations (all methods produced 5 conformations). It is important to note that, regardless of the number of conformations generated for a given task, a successful prediction is acknowledged when the lowest RMSD is no more than 2 Å. As shown in Fig. 2C, KarmaLoop achieved the best performance and improved the success rates by 11.38% and 4.26%, respectively, on the 2 benchmark datasets, compared with the best model NGK with the success rates of 66.13% and 71.95%, respectively. ColabFold and RoseTTAFold exhibited the lowest success rates of only 34.40% and 30.58%, respectively.

To directly compare the performance, the distribution of each method was plotted in Fig. 2D. KarmaLoop showed the lowest medium RMSDs on the CASP13+14 and CASP15 datasets, with 1.41 and 1.31 Å, respectively, as compared to the other methods, and showed much lower RMSDs than the second-best method NGK (1.60 Å) and AlphaFold (1.90 Å). Besides, KarmaLoop achieved the average RMSD of 1.77 Å (±1.43 Å) and 1.95 Å (±2.33 Å), holding a marked leading margin of 42.34% and 34.56% over the next best baseline method NGK on the CASP13+14 and CASP15 datasets (Fig. 2E).

### The impact of loop length on accuracy

**Fig. 3.**
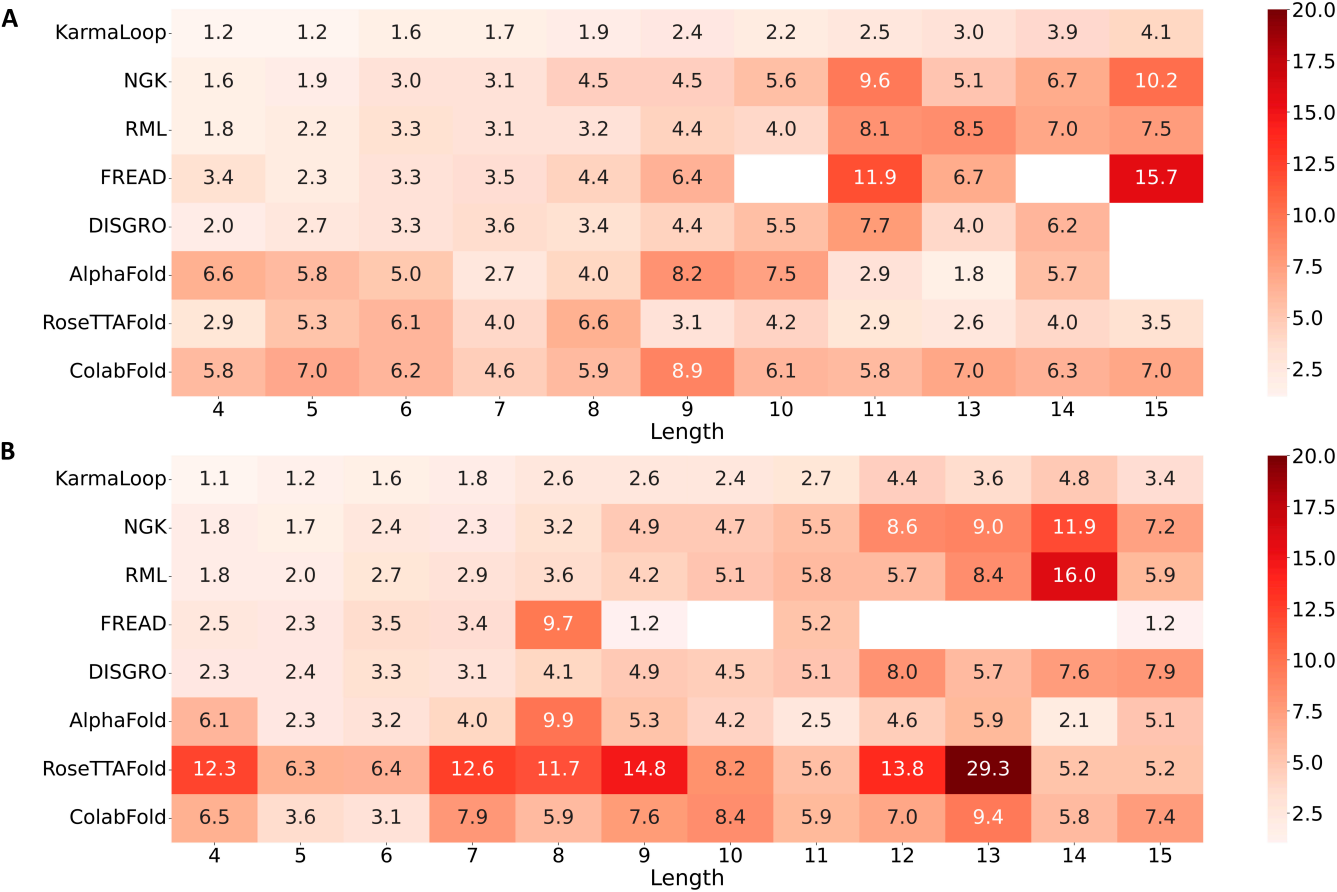
Performance of KarmaLoop and the other tested methods across various loop lengths (loop lengths consist of more than one sample). The performance was tested on (A) the CASP13+14 benchmark dataset and (B) the CASP15 dataset.

We further investigated the influence of loop length on prediction accuracy. As shown in Fig. 1E, KarmaLoop exhibits the possibility to generate satisfactory conformations of loops with varying lengths. Figure 3A and B displays the RMSDs of loop length with more than one sample in the CASP13+14 and CASP15 datasets to avoid statistically inconclusive results. Our results indicated that KarmaLoop outperformed the baseline methods for the majority of length cases (9/11 and 8/12 on the CASP13+14 and CASP15 datasets) and maintained a relatively consistent performance across different loop lengths. In our analysis of over 20,000 proteins from AlphaFold pdb_mmcif dataset, we found that regions comprising no more than 10 residues, predominantly short loops, account for close to 80% of the total missing regions. This finding underscores the critical importance of accurate short loop prediction in protein modeling. Interestingly, AlphaFold and RoseTTAFold fall short in accurately predicting short loops compared with more traditional methods (Fig. 3). In contrast, Karmaloop emerges as a superior method, far surpassing nearly all other methods in short loop precision, with the RMSDs for most loops shorter than 10 residues around 2.0 Å. Besides, the observed increase in RMSD with loop length may be attributed to the fact that the training dataset predominantly consisted of short loops (residues ≤ 10), and long loops (particularly those over 20 residues) were only represented by 0.058% of all the samples (Fig. S1).

### Evaluation of loop modeling efficiency

**Fig. 4.**
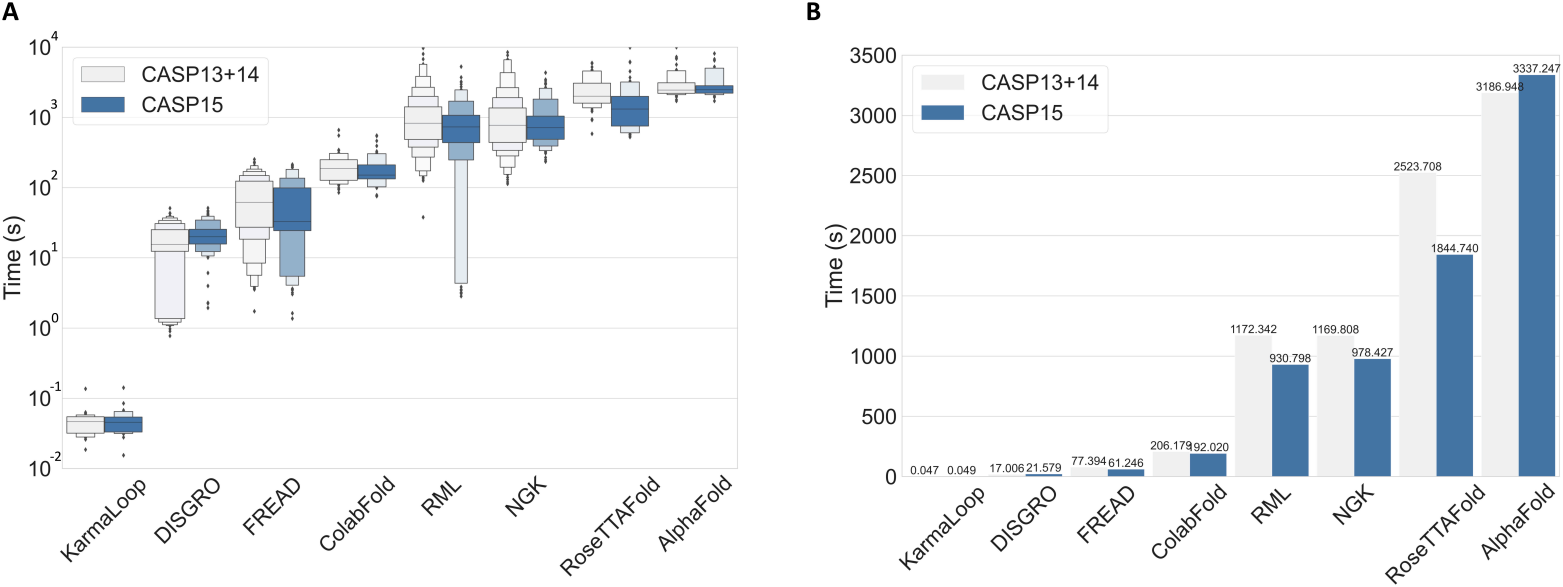
The time cost of each tested method. (A) Time distribution of each method. (B) Average time cost. Further details on the hardware setup can be found in the main text.

Computational efficiency is also a key factor in loop modeling. In this section, the time consumption of each method was evaluated. KarmaLoop was tested on a Tesla V100S GPU, traditional methods (FREAD, DISGRO, NGK, and RML) were run on a single core Intel Xeon Gold 6240R CPU @ 2.40 GHz, and AlphaFold, ColabFold, and RoseTTAFold were evaluated in parallel with 20 cores on the Intel Xeon Gold 6240R CPU @ 2.40 GHz and a Tesla V100S GPU. It is important to emphasize that the results presented by KarmaLoop do not include any postprocessing step, which typically requires approximately 5 s to complete a sample. Figure 4A shows the distribution of time spent for each method, illustrating the remarkable efficiency of KarmaLoop in terms of time cost on each task ranging from 0.015 to 0.14 s. Contrastingly, the time cost of DISGRO ranges from 0.773 to 51.07 s, and the other methods are considerably slower. As shown in Fig. 4B, the average time costs of KarmaLoop on the CASP13+14 and CASP15 datasets are 0.047 and 0.049 s, respectively, displaying a considerable speed advantage over traditional methods. Specifically, the minimum speed advantages of KarmaLoop over DISGRO (17.006 and 21.579 s) are 362× and 440× for CASP13+14 and CASP15, respectively, and the maximum speed advantages of KarmaLoop over RML (1172.342 s) and NGK (978.427 s) are 24,943× and 19,968×, respectively. However, it should be noted that, unlike other methods that only model loop region, AlphaFold, ColabFold, and RoseTTAFold require predicting the entire protein conformation. Although the modeling speed for these methods cannot be directly compared, ColabFold with a customized template database performs faster than AlphaFold and RoseTTAFold. While the reported speedup may vary slightly depending on the hardware used, the 2 to 4 orders of magnitude in speedup should still hold unless these baseline tools have been completely re-engineered to boost their efficiency.

### Prediction of antibody H3 loops

The CDR H3 of antibody is widely acknowledged as the most flexible region and contributes the most to the structural diversity and binding site topography [16]. Consequently, accurate modeling of the H3 conformation is urgently needed. Sphinx, proposed by Marks et al. [22], is a hybrid method that models both general loops and antibody H3 loops with the same architecture but using different fragment databases. In contrast, KarmaLoop consistently employs the same process for H3 modeling.

**Fig. 5.**
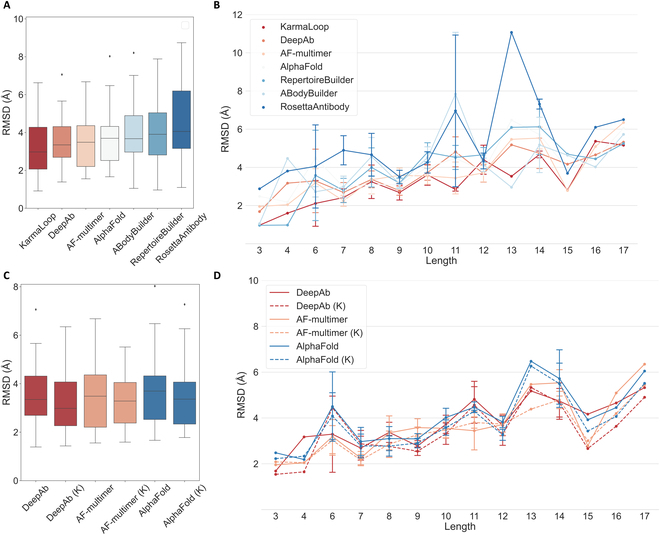
Comparison of the antibody CDR H3 loop prediction accuracy. (A) Distribution of the RMSD values for each method. AF-multimer represents AlphaFold-Multimer. (B) Average RMSDs of different lengths on the tested methods. The error bar shows the Std. of the corresponding length target. (C) Distribution of the RMSD values for each method (K indicates that KarmaLoop refines the H3 loops based on the antibody structures predicted by the corresponding method). (D) Average RMSDs of different lengths on the tested methods.

In this section, we present a comparative analysis of the H3 modeling performance of KarmaLoop against 4 widely used methods (i.e., DeepAb [42], RosettaAntibody-G [59], RepertoireBuilder [60], AbodyBuilder [61], AlphaFold, and AlphaFold-Multimer) for predicting antibody structures on the antibody H3 benchmark. To evaluate the quality of the modeling results, we used the experimentally determined structures from SAbDab as the input. Figure 5A depicts the distribution of the RMSDs between the predicted samples from the baseline methods and the experimentally determined structures. The results demonstrate that KarmaLoop (2.97 and 3.25 Å) outperforms DeepAb (3.35 and 3.49 Å) in terms of the medium and average RMSDs by 0.38 and 0.24 Å, respectively. Figure 5B displays the RMSDs of samples with varying loop lengths, and KarmaLoop exhibits the lowest average RMSD for most lengths of the H3 loop samples.

Next, we assessed the ability of refinement by using the antibody structures predicted by AlphaFold-Multimer, AlphaFold, and DeepAb as the input for KarmaLoop. Notably, different from the version of the models used before, as for the models of the loops in imprecise protein structures, a refined version of the models was used (more details in the “Loop initialization and pocket selection” section). Figure 5C and D illustrates the predictions based on the structures modeled by DeepAb, which can be used to test the capability of KarmaLoop to improve structures under a suboptimal structural situation. The results depicted in Fig. 5 (C and D) demonstrate that KarmaLoop is capable of refining the loop structures with an average RMSD reduction from 3.55 to 3.32 Å, 3.73 to 3.42 Å, and 3.49 to 3.21 Å, and a medium RMSD reduction from 3.45 to 3.23 Å, 3.70 to 3.38 Å, and 3.34 to 2.95 Å for AlphaFold-Multimer, AlphaFold, and DeepAb, respectively. It is worth noting that this improvement is observed across most lengths of the H3 loops, highlighting the consistent ability of KarmaLoop to improve the H3 loop structures regardless of their length. Thus, a universal protocol can be applied for antibody prediction, whereby DeepAb models the entire antibody structures and KarmaLoop reconstructs the H3 structures.

### Multi-preferential conformation prediction

**Fig. 6.**
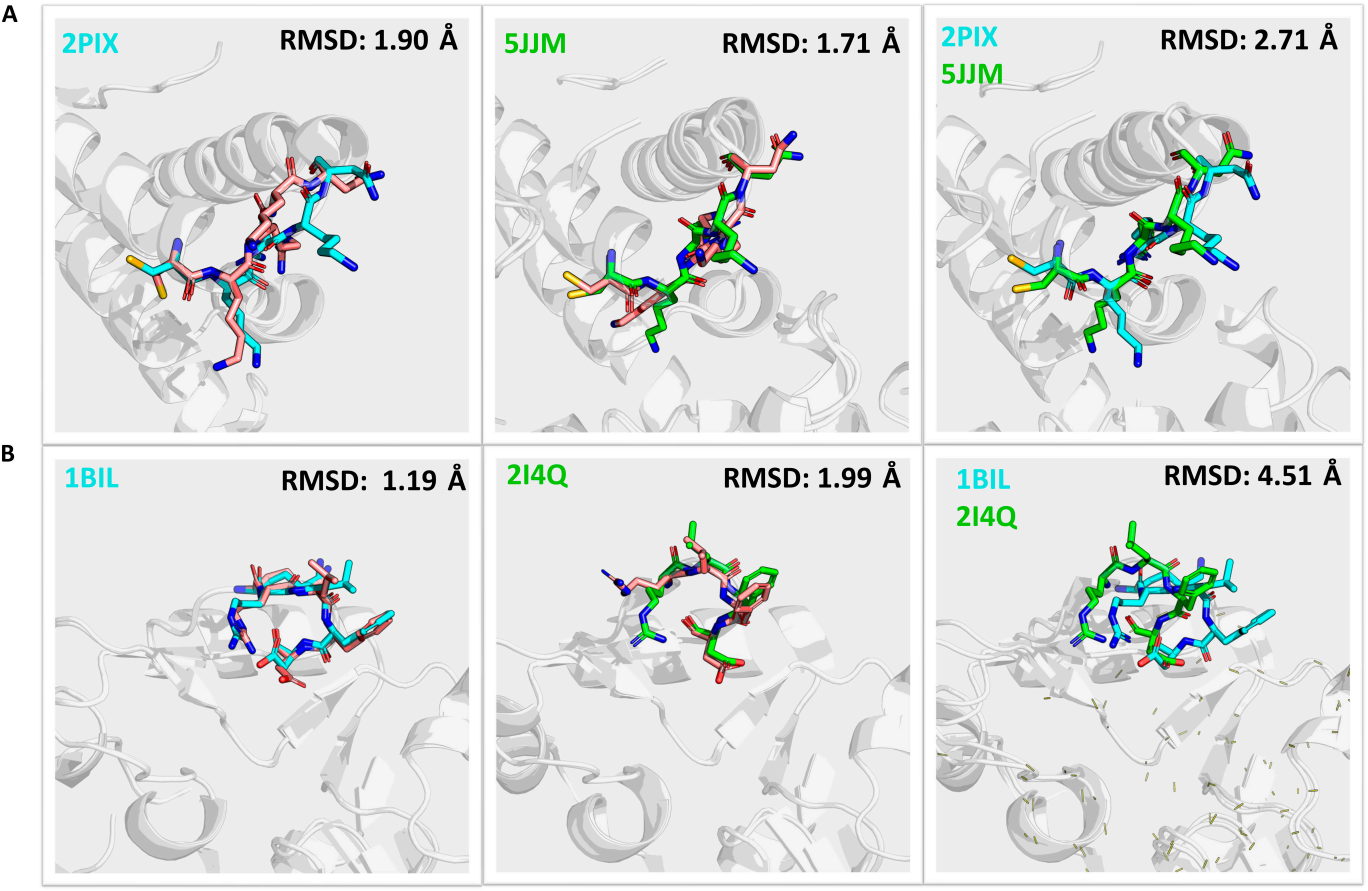
Comparison of KarmaLoop predictions and ground-truth conformations in 2 case studies: (A) AR and (B) Renin. Columns from left to right depict the KarmaLoop predicted conformations (in pink) alongside the 2 experimental-determined ground-truth loop conformations (in blue and green), as well as a comparison between the 2 ground-truth conformations.

In studying the conformational dynamics of protein loops, it is observed that these structures exhibit diverse conformations when binding with different small-molecule ligands, which is very relevant to drug design scenarios. To elucidate the predictive capabilities of KarmaLoop in capturing these multi-conformational states, we investigated the ground-truth structure of 2 well-documented protein families from PDB: the androgen receptor (AR) and Renin, each under distinct conditions and exhibiting unique loop conformations. Specifically, we examined AR in scenarios of small-molecule binding (PDB ID: 2PIX) and homodimer formation (PDB ID: 5JJM); and 2 *holo* forms of Renin in interactions with different small-molecule inhibitors (PDB IDs: 1BIL, 2I4Q). Figure 6 presents a comparative analysis between the conformational predictions made by KarmaLoop and the experimentally determined ground-truth conformations, along with an examination of the discrepancies between 2 sets of ground-truth conformations. In both cases, the loop regions exhibit distinct preferential conformations in complex with different guest molecules, and KarmaLoop demonstrates the possibility of reproducing diverse conformational responses across varying ligand-binding conditions.

## Conclusion

To verify the effectiveness of KarmaLoop, several evaluations and comparisons have been conducted, including general loop modeling accuracy, loop-length impact and compute time, as well as potential applications in antibody H3 prediction. Of note, KarmaLoop achieved a substantial improvement in the average RMSD by more than 2-fold and the medium RMSD by nearly 2-fold. Regarding the success rate, the minimum enhancement of 22.92% and 14.30% in the CASP13+14 and CASP15 benchmark datasets was observed on one conformation generated by each method. Even when compared to other methods that generate multiple conformations, KarmaLoop still achieved a minimum improvement of 11.38% and 4.26%. KarmaLoop is also capable of accurately modeling loops of different lengths and obtained a majority length preponderance against the other methods. Surprisingly, KarmaLoop achieved at least a 362× speed advantage over other traditional methods. In terms of antibody CDR H3 loop modeling, which is considered the most challenging task in antibody structure prediction, KarmaLoop also showed the best performance and maintained its superiority across various lengths. Notably, based on the AlphaFold-Multimer, AlphaFold, and DeepAb modeled antibody structures, KarmaLoop has successfully promoted its accuracy and achieved improvement upon those methods in almost all lengths of H3 loop.

In summary, KarmaLoop has demonstrated the potential to accurately model loops, refine loop conformations based on inaccurate structure environments, and markedly accelerate batch data processing. Besides, a universal protocol was proposed for antibody structure prediction by utilizing DeepAb to model the entire structure of the antibody and KarmaLoop to refine the H3 conformation. Except for an accurate loop modeling paradigm proposed, a large training dataset (0.27 million) and several benchmark datasets have been made available for researchers to further evaluate the prediction accuracy of the general loop and antibody H3. We anticipate that KarmaLoop will accelerate the advancement of protein design, antibody–antigen recognition, and drug discovery and will become an essential tool in the field of biology moving forward.

## Methods

### Architecture overview

Inspired by Jin et al. [62], we consider the loop modeling problem as the docking task and selected pockets for reducing computational resources, which is a common and frequently used protocol for ligand docking [51]. As shown in Fig. 1B, the protein pocket is modeled as a 2D molecular graph, *G_p_*, where nodes represent atoms and edges denote covalent bonds. For the nonloop region, a 3D residue graph (*G_nl_*) is employed, where nodes represent residues. This graph connects each node to its 30 closest neighbors, enhancing the ability to capture long-range interactions more effectively and with less computational demand than an atom-based graph.

Rather than serving as direct inputs for subsequent tasks, the molecular graphs and residue graphs undergo encoding through GT [51–53] and GVP [54] to learn intra-molecular interactions and update node embeddings. Then, for nonloop residues, the node embeddings for atom nodes in the same residue are summed and concatenated to the corresponding residue node embeddings, followed by a feature merging block (FMB) to generate hierarchical node embeddings.

$$H_{p,s}^{1}=\mathrm{GT}\left(H_{p,s}^{0},E_{p,s}^{0}\right)$$ (1)

$$H_{\mathrm{nl},s}^{0.5}=\mathrm{GVP}\left(S_{\mathrm{nl},s}^{0},H_{\mathrm{nl},s}^{0},H_{\mathrm{nl},v}^{0},E_{\mathrm{nl},s}^{0},E_{\mathrm{nl},v}^{0}\right)$$ (2)

$$H_{\mathrm{nl},s,i}^{1}=\mathrm{FMB}\left(\text{concat}\left(H_{\mathrm{nl},s,i}^{0.5},\sum_{j=k}^{k\prime }H_{p,s,j}^{1}\right)\right)$$ (3)

$$H_{l,s}^{1}=H_{p,s}^{1}\left[\text{loop}\_\text{mask}\right]$$ (4)

where *H* denotes the features of nodes and *E* is the characteristics of edges; *S* indicates the type of residue involved; loop mask is a bool array for selecting loop atoms from the whole protein atoms; and *p*, *nl*, *l*, *s*, *v*, *i*, *j*, *k*, and *k*’ on the lower right (i.e., subscripts) denote protein pocket atoms, nonloop residues, loop residues, scalar, vector, the index of residues, the index of atoms, the start index of the atom corresponding to *i*th residue, and the end index of the atom corresponding to *i*th residue, respectively.

Embeddings of nodes $\left(H_{l,s}^{1},H_{\mathrm{nl},s}^{1}\right)$ generated by the encoders, along with the node coordinates $\left(X_{l,s}^{1},X_{\mathrm{nl},s}^{1}\right)$ of loop atoms and nonloop residues, are integrated to create an interaction graph [*G*_*l*,*nl*_ = (*H*_*l*,*nl*_, *E*_*l*,*nl*_, *X*_*l*,*nl*_)]. This graph is designed to encapsulate intermolecular interactions between molecules at both the levels of atoms and residues.

In the procedure for generating poses, the loop conformation, depicted as the coordinates of the loop atom nodes, is initialized by sampling from the normal distribution with means and SDs varying for various tasks (loop reconstruction and loop refinement, details in the “Loop initialization and pocket selection” section). The embeddings for nodes and edges begin from a baseline established by graph normalization and are further processed through an MLP layer (Eq. 5). Subsequently, an EGNN consisting of 8 layers with self-attention is employed to update the embeddings of nodes and edges as well as the positions of the loop atoms. This network architecture is designed to effectively handle interactions both within loop atoms and between loop residue and nonloop atoms (Eq. 6). Inspired by AlphaFold, a recycling approach is adopted to ensure that the EGNN block progressively enhances its ability to refine the structural poses. At the beginning of each recycling iteration, a gating mechanism combines the updated embeddings with the original raw embeddings (Eq. 7).

$$H_{l,\mathrm{nl}}^{0,0}=\text{GraphNorm}\left(H_{l,s},H_{\mathrm{nl},s}\right)$$ (5)

$$H_{l,\mathrm{nl}}^{r,i},E_{l,\mathrm{nl}}^{r,i},X_{l,\mathrm{nl}}^{r,i}=\text{EGNN}\_{\text{Layer}}^{i}\left(H_{l,\mathrm{nl}}^{r,i-1},E_{l,\mathrm{nl}}^{r,i-1},X_{l,\mathrm{nl}}^{r,i-1}\;\right)$$ (6)

$$F_{l,\mathrm{nl}}^{r+1,0}=\text{Gate}\_\text{Block}\left(F_{l,\mathrm{nl}}^{r,8},F_{l,\mathrm{nl}}^{0,0}\right)$$ (7)

In this framework *H* and *E* denote the embeddings of node and edge, respectively; *F* is used to represent a generic embedding; and *l*, *nl*, *r*, and *i* represent loop atoms, nonloop residues, the index of recycling, and the index of EGNN layer, respectively.

Upon obtaining node embeddings, the MDN block is used to predict essential statistical parameters such as the mean, SD, and coefficient of variation. These parameters collectively provide a comprehensive representation of the internodal distance distribution, as outlined in Eq. 8. Following this step, the node-pair embeddings and internodal distances are integrated with the predicted distribution to ascertain the likelihood that an internodal distance mirrors that observed in the crystal conformation (Eq. 9). This resultant probability estimation can then be utilized as a scoring metric to evaluate the appropriateness of the current conformation in subsequent analyses.

$$\mu_{l,\mathrm{nl}},\sigma_{l,\mathrm{nl}},\pi_{l,\mathrm{nl}}=\mathrm{MDN}\_\text{Block}\left(H_{l,\mathrm{nl}}^{1}\right)$$ (8)

$$U_{\left(x\right)}=-\;\sum_{\mathrm{nl}=1}^{\mathrm{NL}}\sum_{l=1}^{L}\text{log}P\left(\left(d_{l,\mathrm{nl}}|h_{l},h_{\mathrm{nl}}\right)\right)=-\text{score}$$ (9)

where *H* represents the node embeddings; *l* and *nl* denote loop atoms and nonloop residues, respectively; and *μ*_*l*,*nl*_, *σ*_*l*,*nl*_, and *π*_*l*,*nl*_ represent the means, SDs, and mixing coefficients, respectively.

### Loop initialization and pocket selection

Selecting the pocket instead of the entire protein can simplify the input of the network and guide KarmaLoop to focus on the relevant structural features that are essential for understanding the interaction between loop and nonloop regions. In our study, we introduce 2 methods for loop initialization and pocket selection.

In cases of loop reconstruction where loop conformations remain undetermined, we calculate the center of mass for each loop residue by uniformly dividing the distance between its anchor residues (those immediately preceding and following the loop region). The initial conformation of each residue is then sampled from a Gaussian distribution, centered on its individual center of mass with an SD of 1.75 Å. The pocket selection similarly relies on the anchor residues, retaining the surface residues within a sphere whose center is the midpoint between the 2 anchor residues (see the “Surface pocket selection” section).

Conversely, for loop conformation refinement, where KarmaLoop is tasked with optimizing the loop conformations that are inaccurately predicted by other algorithms, the conformations are generated from a normal distribution. This distribution has its mean set around the original loop’s center of mass with an SD of 4 Å. Pockets, in this scenario, comprise the residues located within 12 Å of any original loop atom.

### Graph representation

In this research, each protein is depicted as an undirected graph, symbolized by [*G_p_* = (*H_p_*, *E_p_*, *l_p_*)], with nodes corresponding to atoms and edges to covalent bonds. *H_p_* and *E_p_* represent the node features and edge features, while the coordinates of the atoms are represented by the node positions *X_p_*.

To facilitate the prediction of loop conformations through nonbonded interactions, it is essential to establish connections between protein and loop nodes within the model. However, creating full connections between all nonloop and loop nodes is highly resource-intensive, particularly when dealing with a vast array of protein atoms. For obtaining both atom and residue information, we propose to use residues as nodes by merging atom information with the corresponding residues in the nonloop graph, which can also greatly reduce the number of nodes and edges in the graph. Furthermore, residue graphs are capable of encoding the geometric details of each residue while effectively capturing interactions over long distances between loop and nonloop residues, which enhances the prediction of loop conformations. To enhance the prediction of loop conformations, we devise a *K*-nearest neighbor (KNN) graph, denoted as [*G_nl_* = (*H_nl_*, *E_nl_*, *X_nl_*)], setting *K* to 30. In this graph, residues serve as nodes and their coordinates are represented by the coordinates of CA atoms. As detailed in Table S2, the features of both nodes and edges are not limited to scalar features (*H*_*nl*_*s*_, *E*_*nl*_*s*_) but also vector features (*H*_*nl*_*v*_, *E*_*nl*_*v*_), and the GVP is employed to learn the molecular topology and geometric characteristics effectively.

### Graph transformer

In the demonstrated approach for effective representation of protein graphs, GTs have been utilized to learn intramolecular interactions (Fig. 1B). *h*_*p*_*i*_ ∈ *R*^*d_h_*×1^ represents *i*th node feature, and *e*_*p*_*ij*_ ∈ *R*^*d_e_*×1^ represents the edge feature between node *i* and *j*; at the beginning, it was initialed as $h_{p\_i}^{0}$ and $e_{p\_\mathrm{ij}}^{0}$ with *d*-dimension through 2 distinct linear layers:

$$\begin{array}{c} h_{p\_i}^{0}=W_{h}^{0}h_{p\_i}+b_{h}^{0}\\ e_{p\_\mathrm{ij}}^{0}=W_{e}^{0}e_{p\_\mathrm{ij}}+b_{e}^{0} \end{array}$$ (10)

where $W_{h}^{0}\in R^{d\times d_{h}}$, $W_{e}^{0}\in R^{d\times d_{e}}$, and $b_{h}^{0}$, $b_{e}^{0}\in R^{d}$. The node and edge embeddings are first initialized and then processed through a series of 6 graph transformer layers, which produce the final embeddings. Each layer in this stack updates the embeddings through a process of message passing combined with a specialized version of multi-head self-attention (MHA), as detailed in the equations below:

$$q_{p\_i}^{k,l}=W_{Q}^{k,l}\text{Norm}\left(h_{p\_i}^{l}\right)$$ (11)

$$k_{p\_j}^{k,l}=W_{K}^{k,l}\text{Norm}\left(h_{p\_j}^{l}\right)$$ (12)

$$v_{p\_j}^{k,l}=W_{V}^{k,l}\text{Norm}\left(h_{p\_j}^{l}\right)$$ (13)

$$e_{p\_\mathrm{ij}}^{k,l}=W_{E}^{k,l}\text{Norm}\left(e_{p\_\mathrm{ij}}^{l}\right)$$ (14)

$$\begin{array}{c} w_{p\_\mathrm{ij}}^{k,l}={\text{Softmax}}_{\mathrm{j\in N}\left(i\right)}\left(\left(\frac{q_{p\_i}^{k,l}\cdot k_{p\_j}^{k,l}}{\sqrt{d_{k}}}\right)\cdot e_{p\_\mathrm{ij}}^{k,l}\right) \end{array}$$ (15)

$$\begin{array}{c} {\hat{h}}_{p\_i}^{l+1}=h_{p\_i}^{l}+\\ W_{h0}^{l}\text{Dropout}\left({\text{Concat}}_{\mathrm{k\in}1,\ldots ,H},\left.\left(\text{Aggregation}\_{\mathrm{Sum}}_{\mathrm{j\in N}\left(i\right)}\left(w_{p\_\mathrm{ij}}^{k,l}v_{p\_j}^{k,l}\right)\right)\right)\right. \end{array}$$ (16)

$$\begin{array}{c} {\hat{e}}_{p\_\mathrm{ij}}^{\;l+1}=e_{p\_\mathrm{ij}}^{l}+W_{e0}^{l}\text{Dropout}\left({\text{Concat}}_{\mathrm{k\in}1,\ldots ,H}\left(w_{p\_\mathrm{ij}}^{k,l}\right)\right) \end{array}$$ (17)

$$\begin{array}{c} h_{p\_i}^{l+1}={\hat{h}}_{p\_i}^{\;l+1}+W_{h2}^{l}\text{Dropout}\left(\text{SiLU}\left(W_{h1}^{l}\text{Norm}\left({\hat{h}}_{p\_i}^{\;l+1}\right)\right)\right) \end{array}$$ (18)

$$\begin{array}{c} e_{p\_\mathrm{ij}}^{l+1}={\hat{e}}_{p\_\mathrm{ij}}^{\;l+1}+W_{e2}^{l}\text{Dropout}\left(\text{SiLU}\left(W_{e1}^{l}\text{Norm}\left({\hat{e}}_{p\_\mathrm{ij}}^{\;l+1}\right)\right)\right) \end{array}$$ (19)

where $W_{Q}^{k,l}$, $W_{K}^{k,l}$, $W_{V}^{k,l}$, $W_{E}^{k,l}\in R^{d_{k}\times d}$, $W_{h0}^{l}$, $W_{e0}^{l}\in R^{d\times d}$, $W_{h1}^{l}$, $W_{e1}^{k,l}\in R^{2d\times d}$, and $W_{h2}^{l}$, $W_{e2}^{l}\in R^{d\times 2d}$ are learnable parameters from linear layers; *k∈*1, …, *H* represents attention heads number; dimension of each head is denoted by *d_k_*; *j∈N*(*i*) denotes the set of nodes adjacent to node *i*; *Norm* refers to the application of batch normalization; *Concat* involves the operation of concatenation; *Dropout* operation involves randomly omitting units during training to prevent overfitting; *SiLU* refers to a specific activation function; *Aggregation*_*Sum*_*j∈N*(*i*)_ implies the summing up of messages from edges that link node *i* with its neighbors *j*; and *Softmax*_*j∈N*(*i*)_ denotes the SoftMax function applied across the neighbors *j*.

### Geometric Vector Perceptrons

In the KarmaLoop implementation, GVPs serve as the nonloop residue encoder. They utilize node embeddings updated according to topological connections and geometric data within and between residues. The fundamental component, known as the gvp layer, processes scalar features *f_s_* ∈ *R^d^* and vector features *f_v_* ∈ *R*^*d*×3^. The operation within the *l*th layer of this system can be described as follows:

$$f_{v1}^{l}=W_{v0}^{l}\;f_{v}^{l}$$ (20)

$$f_{v2}^{l}=W_{v1}^{l}f_{v1}^{l}$$ (21)

$$S_{v1}^{l}={\left\|\;f_{v1}^{l}\right\|}_{2}\left(\mathrm{row}\;\text{wise}\right)$$ (22)

$$S_{v2}^{l}={\left\|\;f_{v2}^{l}\right\|}_{2}\left(\mathrm{row}\;\text{wise}\right)$$ (23)

$$f_{\mathrm{sv}}^{l}=\text{Concat}\left(f_{s}^{l},S_{v1}^{l}\right)$$ (24)

$${\hat{f}}_{s}^{l}=W_{\mathrm{sv}}^{l}\;f_{\mathrm{sv}}^{l}+b_{\mathrm{sv}}^{l}$$ (25)

$$f_{s}^{l+1}=\sigma_{s}\left({\hat{f}}_{s}^{l}\right)$$ (26)

$$f_{v}^{l+1}=\sigma_{v}\left(S_{v2}^{l}\right)⊙f_{v2}^{l}\;\left(\mathrm{row}\;\text{wise multiplication}\right)\;$$ (27)

where $W_{v0}^{l}\in R^{d_{v1}\times d_{v0}}$, $W_{v1}^{l}\in R^{d_{v2}\times d_{v1}}$, and $W_{\mathrm{sv}}^{l}\in R^{d_{s1}\times \left(d_{s0}+d_{v1}\right)}$ are learnable parameters; activation functions are denoted as *σ_s_* and *σ_v_*. Initially, sequence information is processed through an embedding layer and then concatenated with additional scalar node features before entering the gvp layers:

$$h_{\mathrm{seq}}=\text{Embedding}\left(\text{Sequence}\right)$$ (28)

$$h_{s}=\text{Concat}\left(h_{s0},h_{\mathrm{seq}}\right)$$ (29)

In the architecture of the embedding layer, the dimension of the word table is structured as (*d_seq_*, *d_seq_*) and *h*_*s*0_*∈R*^*d*_*hs*0_^. Following this, both node features and edge features are fed into an initialization module, which includes a layer normalization and a gvp layer.:

$$\left(h_{s1},h_{v1}\right)=\mathrm{gvp}\left(\text{LayerNorm}\left(h_{s},h_{v}\right)\right)$$ (30)

$$\left(e_{s1},e_{v1}\right)=\mathrm{gvp}\left(\text{LayerNorm}\left(e_{s},e_{v}\right)\right)$$ (31)

where *h_s_∈R*^*d_seq_*+*d*_*hs*0_^, *h_v_∈R*^*d*_*hv*0_×3^, *h*_*s*1_*∈R*^*d*_*hs*1_^, *h*_*v*1_*∈R*^*d*_*hv*1_×3^, *e_s_∈R*^*d*_*es*0_^, *e_v_∈R*^*d*_*ev*0_×3^, *e*_*s*1_*∈R*^*d*_*es*1_^, and *e*_*v*1_*∈R*^*d*_*ev*1_×3^. Following the initial setup, both node and edge attributes are fed into a sequence of GVPConv layers, where the process incorporates 2 stages of GVP layers for message transmission. The formulas defining the GVPConv layer operations are presented below:

$$m_{s\_\mathrm{ij}}^{l}=\text{Concat}\left(h_{s\_i}^{l},e_{s\_\mathrm{ij}}^{l},h_{s\_j}^{l}\right)$$ (32)

$$m_{v\_\mathrm{ij}}^{l}=\text{Concat}\left(h_{v\_i}^{l},e_{v\_\mathrm{ij}}^{l},h_{v\_j}^{l}\right)$$ (33)

$$\left(m_{s\_\mathrm{ij}\_1}^{l},m_{v\_\mathrm{ij}\_1}^{l}\right)=\mathrm{gvp}\left(m_{s\_\mathrm{ij}}^{l},m_{v\_\mathrm{ij}}^{l}\right)$$ (34)

$$\left(m_{s\_\mathrm{ij}\_2}^{l},m_{v\_\mathrm{ij}\_2}^{l}\right)=\mathrm{gvp}\left(m_{s\_\mathrm{ij}\_1}^{l},m_{v\_\mathrm{ij}\_1}^{l}\right)$$ (35)

$$\left(m_{s\_\mathrm{ij}\_3}^{l},m_{v\_\mathrm{ij}\_3}^{l}\right)=\mathrm{gvp}\left(m_{s\_\mathrm{ij}\_2}^{l},m_{v\_\mathrm{ij}\_2}^{l}\right)$$ (36)

$$\begin{array}{c} \left({\hat{h}}_{s\_j}^{l},{\hat{h}}_{v\_j}^{l}\right)=\text{Aggregation}\_{\text{Mean}}_{\mathrm{i\in N}\left(j\right)}\;\left(m_{s\_\mathrm{ij}\_3}^{l},m_{v\_\mathrm{ij}\_3}^{l}\right) \end{array}$$ (37)

$$\begin{array}{c} \left({\hat{f}}_{s\_j\_0}^{l},{\hat{f}}_{v\_j\_0}^{l}\right)=\\ \text{LayerNorm}\left(h_{s\_j}^{l}+\text{Dropout}\right.\left.\left({\hat{h}}_{s\_j}^{l}\right),h_{v\_j}^{l}+\text{Dropout}\left({\hat{h}}_{v\_j}^{l}\right)\right) \end{array}$$ (38)

$$\left({\hat{f}}_{s\_j\_1}^{l},{\hat{f}}_{v\_j\_1}^{l}\right)=\mathrm{gvp}\left({\hat{f}}_{s\_j\_0}^{l},{\hat{f}}_{v\_j\_0}^{l}\right)$$ (39)

$$\left({\hat{f}}_{s\_j\_2}^{l},{\hat{f}}_{v\_j\_2}^{l}\right)=\mathrm{gvp}\left({\hat{f}}_{s\_j\_1}^{l},{\hat{f}}_{v\_j\_1}^{l}\right)$$ (40)

$$\begin{array}{c} \left(h_{s\_i}^{l+1},h_{v\_j}^{l+1}\right)=\\ \text{LayerNorm}\left(h_{s\_j}^{l},+,\text{Dropout}\right.\left.\left({\hat{f}}_{s\_j\_2}^{l}\right),h_{v\_j}^{l}+\text{Dropout}\left({\hat{f}}_{v\_j\_2}^{l}\right)\right) \end{array}$$ (41)

In Eqs. 34, 35, and 39, the scalar and vector features utilize ReLU and sigmoid activation functions, respectively. Contrarily, other gvp layers operate without activation functions; $m_{s\_\mathrm{ij}}^{l}\in R^{2d_{\mathrm{hs}1}+d_{\mathrm{es}1}}$, $m_{v\_\mathrm{ij}}^{l}\in R^{\;\left(2d_{\mathrm{hv}1}+d_{\mathrm{ev}1}\right)\times 3}$, $m_{s\_\mathrm{ij}\_1}^{l}$, $m_{s\_\mathrm{ij}\_2}^{l}$, $m_{s\_\mathrm{ij}\_3}^{l}$, ${\hat{h}}_{s\_j}^{l}$, ${\hat{f}}_{s\_j\_0}^{l}$, ${\hat{f}}_{s\_j\_2}^{l}$ and $h_{s\_i}^{l+1}\in R^{d_{\mathrm{hs}1}}$, $m_{v\_\mathrm{ij}\_1}^{l}$, $m_{v\_\mathrm{ij}\_2}^{l}$, $m_{v\_\mathrm{ij}\_3}^{l}$, ${\hat{h}}_{v\_j}^{l}$, ${\hat{f}}_{v\_j\_0}^{l}$, ${\hat{f}}_{v\_j\_2}^{l}$ and $h_{v\_j}^{l+1}\in R^{d_{\mathrm{hv}1}\times 3}$, ${\hat{f}}_{s\_j\_1}^{l}\in R^{4d_{\mathrm{hs}1}}$, and ${\hat{f}}_{v\_j\_1}^{l}\in R^{\left(2d_{\mathrm{hv}1}\right)\times 3}$; and *Aggregation*_*Mean*_*i∈N*(*j*)_ indicates the process of averaging the messages across the edges that link node *j* to its adjacent nodes *i*.

### Hierarchical representation

One of the innovations of KarmaLoop is the hierarchical representation of protein residues and atoms. By adopting both the advantage of atom-level representation (offering detailed descriptions but demanding high resource) and residue-level representation (requiring lower resource but providing less details) of protein, hierarchical representation achieves an effective balance between comprehensive protein characterization with reduced resource demands. As depicted in Fig. 1C, the scalar embeddings *h*_*p*_*i*_ and *h*_*nl*_*i*_ generated from the GVP block and GT block for the protein atoms and nonloop residues are merged. The atom node features are summed and concatenated with the corresponding residue features followed by linear layers and an activation layer for feature fusion.

$$m_{\mathrm{nl}\_i}=\text{Concat}\left(h_{\mathrm{nl}\_i},\sum_{j=k}^{k\prime }h_{p\_j}\right)$$ (42)

$$m_{\mathrm{nl}\_i}=\mathrm{MLP}\left(\text{LaekyReLU}\left(\mathrm{MLP}\left(m_{\mathrm{nl}\_i}\right)\right)\right)$$ (43)

where *p*, *nl*, *i*, *j*, *k*, and *k*’ on the lower right (i.e., subscripts) denote protein atoms, nonloop residues, the index of nonloop residues, the index of nonloop atoms, the start index of the atom corresponding to *i*th residue, and the end index of the atom corresponding to *i*th residue, respectively.

### Constructing interaction graph

For predicting loop conformations, we design an interaction graph [*G*_*l*,*nl*_ = (*H*_*l*,*nl*_, *E*_*l*, *nl*_,*X*_*l*,*nl*_)] derived from the overall protein atom graph *G_p_* and nonloop residue graph *G_nl_*. Previously, we constructed the loop graph and nonloop graph, with edges connecting nodes within the same region. However, it is crucial to establish connections between these regions. Therefore, we construct an interaction graph, which facilitates the exploration of interactions between loop and nonloop regions, thereby deepening our comprehension of their interconnected dynamics. We take nonloop residues and loop atoms as nodes. Then, we fully connect nonloop and loop nodes and consider the global interactions for accurate generation of loop conformations. Initial node features *h*_0_ ∈ *R*^*d*_*h*0_^ are obtained from the GT encoder’s output and merged feature from the output of merge block for loop atoms and nonloop residues, respectively. Edge features *e*_0_ ∈ *R*^*d*_*e*0_^ are generated through one-hot encoding of edge types and the distances between nodes, with actual distances used for intra-residue and intra-loop edges and −1 for others. Moreover, the coordinates of nonloop nodes *x*_0_ ∈ *R*^*n*×3^ are directly inherited from the protein graphs, whereas the loop conformation are initialized randomly.

### EGNN block

In this research, we incorporate self-attention mechanisms into the message-passing process of the E(n) equivariant model, EGNN, which is noted for its rapid processing capabilities in dynamical systems. Unlike traditional GNNs, the integration of self-attention, akin to transformations seen in RTMScore [51], greatly enhances the representational capacities of GNNs. Additionally, to enhance accuracy, we implement a recycling mechanism inspired by AlphaFold that compels the EGNN layer to predict atomic movements based on the current predicted conformation.

Initially, the scalar node embeddings for proteins and loops, *h*_0_, are set using graph normalization techniques, with updates processed via GVP and GT blocks. The initialization of edge features, *e*_0_, is updated through a dedicated linear layer:

$$h_{1}=\text{GraphNorm}\left(h_{0}\right)$$ (44)

$$e_{1}=W_{e\_\text{init}}e_{0}+b_{e\_\text{init}}$$ (45)

where *h*_0_, *h*_1_ and *e*_1_*∈R^d_h_^*, *e*_0_*∈R^d_e_^*; *W*_*e*_*init*_*∈R*^*d_h_* × *d_e_*^ and *b*_*e*_*init*_*∈R^d_h_^* are learnable within the linear layer.

The primary building block for updating loop coordinates employs a sequence of 8 sequentially stacked EGNN layers. The message-passing mechanism in the *l*th layers of EGNN layer is depicted as follows:

$${\left(q_{i}^{k,l}\right)}_{\mathrm{k\in}1,\ldots ,H}=W_{Q}^{l}h_{i\_1}^{l}+b_{Q}^{l}$$ (46)

$${\left(k_{j}^{k,l}\right)}_{\mathrm{k\in}1,\ldots ,H}=W_{K}^{l}h_{j\_1}^{l}+b_{K}^{l}$$ (47)

$${\left(v_{j}^{k,l}\right)}_{\mathrm{k\in}1,\ldots ,H}=W_{V}^{l}h_{j\_1}^{l}+b_{V}^{l}$$ (48)

$$e_{\mathrm{ij}}^{l}=\text{Concat}\left(e_{\mathrm{ij}\_1}^{l},{\left\|x_{i}^{l}-x_{j}^{l}\right\|}_{2}\right)$$ (49)

$$m_{\mathrm{ij}}^{l}=W_{m2}^{l}\left(\text{LeakyReLU}\left(\text{Dropout}\left(W_{m1}^{l}e_{\mathrm{ij}}^{l}+b_{m1}^{l}\right)\right)\right)+b_{m2}^{l}$$ (50)

$$\begin{array}{c} {\left(k_{\mathrm{ij}}^{k,l}\right)}_{\mathrm{k\in}1,\ldots ,H}={\text{Concat}}_{\mathrm{k\in}1,\ldots ,H}\left(k_{j}^{k,l}\right)⊙m_{\mathrm{ij}}^{l} \end{array}$$ (51)

$$w_{\mathrm{ij}}^{k,l}=\left(q_{i}^{k,l}⊙k_{\mathrm{ij}}^{k,l}\right)/\sqrt{d_{k}}$$ (52)

$$\alpha_{\mathrm{ij}}^{k,l}={\text{Softmax}}_{\mathrm{j\in N}\left(i\right)}\left({\left\|w_{\mathrm{ij}}^{k,l}\right\|}_{2}\right)$$ (53)

$$\begin{array}{c} {\hat{h}}_{i\_1}^{l}=\\ \text{Dropout}\left(W_{h}^{l},\left({\text{Concat}}_{\mathrm{k\in}1,\ldots ,H},\left.\left.\left(\text{Aggregation}\_{\mathrm{Sum}}_{\mathrm{j\in N}\left(i\right)}\;\left(w_{\mathrm{ij}}^{k,l}⊙v_{j}^{k,l}\right)\right)\right)+b_{h}^{l}\right)\right.\right. \end{array}$$ (54)

$$h_{i\_1}^{l+1}=\text{Gate}\_\text{Block}\left(h_{i\_1}^{l},{\hat{h}}_{i\_1}^{l}\right)$$ (55)

$$e_{\mathrm{ij}\_1}^{l+1}=W_{e}^{l}{\text{Concat}}_{\mathrm{k\in}1,\ldots ,H}\left(\alpha_{\mathrm{ij}}^{k,l}\right)+b_{e}^{l}$$ (56)

xil+1=Coords_Update_Blockwijk,lkϵ1,…,H,jϵNi,xil,xjljϵNi (57)

In linear layers $\alpha_{\mathrm{ij}}^{k,l}\in R^{1}$; $h_{i\_1}^{l}$, $h_{j\_1}^{l}$, $e_{\mathrm{ij}\_1}^{l}$, $e_{\mathrm{ij}}^{l}$, $m_{\mathrm{ij}}^{l}$, ${\hat{h}}_{i\_1}^{l}$, $h_{i\_1}^{l+1}$, and $e_{\mathrm{ij}\_1}^{l+1}\in R^{d_{h}}$; $q_{i}^{k,l}$, $k_{j}^{k,l}$, $k_{\mathrm{ij}}^{k,l}$, $v_{j}^{k,l}$, $w_{\mathrm{ij}}^{k,l}\in R^{d_{k}}$; $W_{Q}^{l}$, $W_{K}^{l}$, $W_{V}^{l}$, $W_{m2}^{l}$, $W_{h}^{l}$, and $W_{e}^{l}\in R^{d_{h}\times d_{h}}$, $W_{m1}^{l}\in R^{d_{h}\times \left(d_{h}+1\right)}$, $b_{Q}^{l}$, $b_{K}^{l}$, $b_{V}^{l}$, $b_{m1}^{l}$, $b_{m2}^{l}$, $b_{h}^{l}$, and $b_{e}^{l}\in R^{d_{h}}$ are learnable; *k∈*1, …, *H* indicates the total number of attention heads; each head’s dimension, *d_k_*, is the quotient of *d_h_* by *H*; *j∈N*(*i*) indicates the nodes adjacent to node *i*; *Concat* refers to the operation of concatenation; Dropout is an operation used to prevent overfitting; *LeakyReLU* represents a variant of activation functions; *Aggregation*_*Sum*_*j∈N*(*i*)_ describes the process of aggregating messages from edges linking node *i* to its adjacent nodes *j*; and *Softmax*_*j∈N*(*i*)_ is the SoftMax function applied to the neighboring nodes *j*.

The loop node coordinates update was implemented by the Coords_Update_Block:

$$∆\overset{⃑}{x_{\mathrm{ij}}^{l}}=x_{i}^{l}-x_{j}^{l}$$ (58)

$$∆\overset{⃑}{x_{\mathrm{ij}}^{l}}=∆\overset{⃑}{x_{\mathrm{ij}}^{l}}/{\left\|∆\overset{⃑}{x_{\mathrm{ij}}^{l}}\right\|}_{2}$$ (59)

$$\begin{array}{c} ∆x_{\mathrm{ij}}^{k,l}=\\ ∆\overset{⃑}{x_{\mathrm{ij}}^{l}}\cdot \left(W_{x2}^{l}\left(\text{LeakeyReLU}\left.\left.\left(\text{Dropout}\left(W_{x1}^{l}w_{\mathrm{ij}}^{k,l}+b_{x1}^{l}\;\right)\;\right)\;\right)+b_{x2}^{l}\;\right)\;\right.\;\right. \end{array}$$ (60)

$$∆x_{\mathrm{ij}}^{l}=W_{H}^{l}{\text{Concat}}_{\mathrm{k\in}1,\ldots ,H}\left(∆x_{\mathrm{ij}}^{k,l}\right)$$ (61)

$$\begin{array}{c} ∆x_{i}^{l}=\text{Aggregation}\_{\mathrm{Sum}}_{\mathrm{j\in N}\left(i\right)}\left(∆x_{\mathrm{ij}}^{l}\right) \end{array}$$ (62)

$$x_{i}^{l+1}=x_{i}^{l}+∆x_{i}^{l}$$ (63)

where $x_{i}^{l}$, $x_{j}^{l}$, $\text{∆}\overset{⃑}{x_{\mathrm{ij}}^{l}}$, $\text{∆}x_{\mathrm{ij}}^{k,l}$, $\text{∆}x_{i}^{l}$, and $x_{i}^{l+1}{\in R}^{1\times 3}$; $W_{\;x1}^{l}\in R^{\left(d_{k}/2\right)\times d_{k}}$, $W_{\;x2}^{l}\epsilon R^{1\times \left(d_{k}/2\right)}$, $W_{\;H}^{l}\epsilon R^{1\times \left(H\right)}$, $b_{\;x1}^{l}{\in R}^{\left(d_{k}/2\right)}$, and $b_{\;x2}^{l}{\in R}^{1}$.

Gate_Block serves as a foundational component for residual connections within EGNN layers and initiates each cycle of recycling.

$$g=\text{Sigmoid}\left(\text{Dropout}\left(W_{g}\text{Concat}\left({\hat{h}}_{\mathrm{new}},h_{\mathrm{old}},{\hat{h}}_{\mathrm{new}}-h_{\mathrm{old}}\;\right)+b_{g}\right)\right)$$ (64)

$$h_{\mathrm{new}}=\text{GraphNorm}\left(g⊙{\hat{h}}_{\mathrm{new}}+h_{\mathrm{old}}\right)$$ (65)

where *h_old_*, ${\hat{h}}_{\mathrm{new}}$, *g*, and *h_new_∈R^d_h_^*; *W_g_∈R*^*d_h_* × *d_h_*^ and *b_g_∈R^d_h_^* are learnable parameters.

### Mixture density network

The MDN block is used to learn the prior knowledge of distance. The process starts by sequentially concatenating the node embeddings from nonloop residues (*h_nl_*) and loop atoms (*h_l_*) produced by GVP block and GT block, respectively. This concatenated output is then passed through a linear layer, followed by batch normalization, an ELU activation function, and a dropout layer to enhance the capturing of interactions. Subsequently, 3 distinct linear layers are employed to compute the parameters: means *μ*_*p*,*c*_, SDs *σ*_*p*,*c*_, and mixing coefficients *π*_*p*,*c*_. These parameters are vital for defining a mixture density model that captures a distribution of *n* different distance distributions for each pair between nonloop and loop nodes:

$$\begin{array}{c} h_{\mathrm{nl},l}=\\ \text{Dropout}\left(\mathrm{ELU},\left(\text{BatchNorm},\left.\left.\left(W_{p,c}\text{Concat}\left(h_{\mathrm{nl}},h_{l}\right)+b_{\mathrm{nl},l}\right)\right)\right)\right.\right. \end{array}$$ (66)

$$\mu_{\mathrm{nl},l}=\mathrm{ELU}\left(W_{\;\mu }h_{\mathrm{nl},l}+b_{\;\mu }\right)+1$$ (67)

$$\sigma_{\mathrm{nl},l}=\mathrm{ELU}\left(W_{\;\sigma }h_{\mathrm{nl},l}+b_{\;\sigma }\right)+1.1$$ (68)

$$\pi_{\mathrm{nl},l}=\text{Softmax}\left(W_{\;\pi }h_{\mathrm{nl},l}+b_{\;\pi }\right)$$ (69)

where *W*_*nl*,*l*_*∈R*^*d*_*nl*,*l*_×2*d_h_*^, *W_μ_*, *W_σ_*, *W_π_∈R*^*n*×*d*_*nl*,*l*_^, *b*_*p*,*c*_*∈R*^*d*_*nl*,*l*_^, *b_μ_*, *b_σ_*, and *b_π_∈R^n^* are learnable parameters of linear layers; *h_nl_* and *h_l_∈R^d_h_^*.

In our approach to modeling proteins, we utilize both residue-level and atom-level representations. For determining the proximity between loop atoms and nonloop atoms, we select the minimum distance between each loop atom and every atom in a residue as the key metric for assessing the distance between loop and nonloop nodes.

### Postprocessing

After KarmaLoop predicted the conformation, an optional force field (FF) optimization was implemented to remove unfavorable steric clashes and generate high-quality loop conformations. OpenMM [63] was deployed for energy minimization by using the ff14SB FF to optimize the predicted conformation. To prevent excessive changes to the protein, we applied a harmonic potential that restrains the backbone atoms (N, CA, C, and CB) to their original locations. Finally, we ran energy minimization on an A100 GPU and saved the optimized protein structure to a new PDB file (with an average speed of about 5 s per loop). Additionally, high tolerance was selected to make sure that not too much expensive computational cost was used, and in practical use, the tolerance can be selected automatically.

### Surface pocket selection

The surface pocket selection includes 2 processes.

#### Radius determination

Initially, a midpoint is determined between 2 anchor residues (the nonloop residues immediately before and after the loop), serving as the preliminary center of the pocket. Subsequently, 2 specific radii are calculated:

a. Half the distance between the centers of mass of the 2 anchor residues.

b. A value derived from doubling the number of loop residues, expressed in Å, with a maximum limit of 16 Å.

From these, the larger radius is chosen for the pocket.

#### Residue reduction

The selected radius dictates one of 2 scenarios (Fig. S2):

a. if the radius is greater than 20 Å, pseudo-loop conformations are sampled uniformly using a linear interpolation method between the 2 anchor residue conformations. The residues located within 12 Å of these pseudo-loop conformations are included in the pocket.

b. In other cases, this radius is employed to define a smaller sphere, *SS*, centered at the previously determined midpoint. Nonloop residues located within *SS* are chosen as the initial pocket. A larger sphere, *BS*, is subsequently generated using an extended radius (initial radius + 12 Å), aiming to encompass all potential interacting residues. However, the *BS*-defined pocket may incorporate many residues distant from the loop–nonloop interaction interface, which may have minimal impact on loop conformation prediction. Hence, we reduced the pocket residues by removing the residues only selected by *BS* but forming similar residue-midpoint directions to these selected by *SS*. To further enhance the pocket, the residues located within 5 to 7 Å of the already selected pocket residues are also included.

### Benchmark dataset

The biennial Critical Assessment of protein Structure Prediction (CASP) is a worldwide contest for protein structure prediction and is also commonly used for loop modeling assessment [17,20,23,64]. The general loop benchmark consists of 2 datasets: CASP13+14 and CASP15 monomer datasets. CASP13+14 is the combination of CASP13 and CASP14, which contains 549 loop structures from 52 proteins (the unmatched structure and sequence sample were removed). CASP15 containing 430 loop structures from 48 proteins was collected from the latest CASP competition. Then, the pocket sequence similarity was calculated by BLAST+ [65] (*blastp* module), and the sequences with no more than 50% similarity between CASP13+14/CASP15 and the training data were selected as the loop benchmark. Finally, 289 loop structures from 39 proteins in CASP13+14 and 304 loop structures from 41 proteins in CASP15 were selected as the benchmark. The antibody H3 benchmark derived from DeepAb is the combination of the RosettaAntibody and therapeutic benchmark [66], and comprises 92 antibody CDR H3 loop structures from 92 antibody complex structures. According to the standard of the pocket sequence similarity of less than 90% and H3 sequence similarity of less than 50%, we finally got 50 antibodies. The distribution of the loop lengths of those benchmarks is depicted in Fig. S1.

### Training dataset

In this study, the training data were collected from PDB and Structural Antibody Database (SAbDab) [67]. The protein data from PDB were filtered by PISCES [68] using the following criteria: solved by x-ray crystallography, sequence identity ≤ 90%, resolution < 3.0 Å, *R* factor ≤ 0.25, and containing no DNA, RNA, and UNK molecules. The nonsecondary structure of PDB data was defined by DSSP [69], and we defined loops as regions connecting 2 secondary structures that consist of at least 4 residues. The antibody dataset was selected to be the same as that of DeepAb. The training and validation datasets were created using random stratified sampling according to the loop length in a 9:1 ratio. The antibody data were annotated using the Chothia numbering scheme [70]. Specifically, the training data contain the CDR L1, L2, L3, H1, H2, and H3 regions, and each type of CDRs was also split following the 9:1 ratio, according to loop length. Finally, the total numbers of the data for training and validation are 250,257 and 27,832, respectively.

### Dataset preprocessing

All the protein data used in this work were downloaded from PDB, SAbDab, and CASP. Water molecules were subsequently removed from the proteins, and the loop regions with nonstandard amino acid residues, missing residues, and incorrect valence bonds were removed. The nonloop pockets were selected in the way mentioned in the “Loop initialization and pocket selection” section.

### Training protocol

In response to different application requirements, we formulated 2 specialized training variants for KarmaLoop. For the loop reconstruction task, KarmaLoop was trained on surface pockets by initializing loop conformations without reliance on the ground-truth data. Conversely, when optimizing loop conformations derived from less precise frameworks, such as those predicted by AlphaFold, the training of KarmaLoop focused on pockets located within 12 Å of the raw loop conformations. These conformations were centered around the raw loop’s center of mass.

In the training process of the model, it is hypothesized that the MDN block’s ability to fit distributions of distances between nonloop and loop node pairs enhances the model’s efficiency in constraining the search space for stable conformations, thereby facilitating the identification of the most optimal conformation. For the purpose of predicting conformations, we initially train the MDN block within the KarmaLoop framework using updated node embeddings from encoders and actual loop conformation data as inputs. This training employs an MDN-specific loss function *L_MDN_* alongside 2 auxiliary cross-entropy loss functions (*L_atom_*, *L_bond_*), targeting different aspects of the model:

$$L=L_{\mathrm{MDN}}+0.001\times L_{\text{atom}}+0.001\times L_{\text{bond}}$$ (70)

$$\begin{array}{c} \begin{array}{c} L_{\mathrm{MDN}}=-\text{log}P\left(\left(d_{l,\mathrm{nl}}|h_{l},h_{\mathrm{nl}}\right)\right)\\ \;=-\text{log}\sum_{n=1}^{10}\pi_{l,\mathrm{nl},n}N\left(\left(d_{l,\mathrm{nl}}|\mu_{l,\mathrm{nl},n},\sigma_{l,\mathrm{nl},n}\right)\right) \end{array} \end{array}$$ (71)

where *μ*_*l*,*nl*,*n*_, *σ*_*l*,*nl*,*n*_, and *π*_*l*,*nl*,*n*_ represent mean, SDs, and the mixing coefficients of *n*th distance distribution; *h_l_*, *h_nl_*, and *d*_*l*,*nl*_ represent the node embeddings of loop atom nodes, nonloop residue nodes, and the distance between loop and nonloop nodes, respectively. The model was optimized using the Adam optimizer with a batch size of 64, a learning rate of 1 × 10^−3^, and a weight decay of 1 × 10^−5^. Training ceases when the validation loss rises for 70 consecutive epochs. At this stage, both the GT and GVP are partially attuned to the distance distributions. Subsequently, we initiate training of the EGNN module and the previously developed scoring module, utilizing the same dataset partitioning technique as in the initial training of the scoring module. We integrate the conformation predicted module’s loss function into Eq. 1 with a coefficient of 1, correlating it to the RMSD between the predicted loop conformations and their actual counterparts. The training hyperparameters remain unchanged except for a reduction in the learning rate to 1 × 10^−4^ and the removal of weight decay.

$$L=L_{\text{rmsd}}+L_{\mathrm{MDN}}+0.001\times L_{\text{atom}}+0.001\times L_{\text{bond}}$$ (72)

$$L_{\text{rmsd}}=\text{RMSD}\left(x_{l}^{\text{pred}},x_{l}^{\text{label}}\right)=\sqrt{\frac{\sum_{n=1}^{N}{\left(x_{l,n}^{\text{pred}}-x_{l,n}^{\text{label}}\right)}^{2}}{N}}$$ (73)

where *N* denotes the loop node number and *n* represents the index of the loop nodes.

### Multi-conformation KarmaLoop

In our study, we observed that initializing the loops with different SDs led to diverse loop conformations, which partially captured the loop’s conformational flexibility. Upon evaluating various SDs for loop initialization, we determined that a deviation of 1.75 Å, identical to the training set, yielded the highest success rate for single conformation. In contrast, a deviation of 7 Å was optimal for achieving success in multi-conformation predictions. Consequently, we used 1.75 Å for single-conformation generation and 7 Å for multi-conformation generation.

### Evaluation methods

To assess the performance of KarmaLoop, we selected well-established traditional and DL methods renowned for their precision or efficiency. This choice was grounded in our thorough evaluation of loop modeling methods, as detailed in our previous research [71]. The general protein loop modeling methods that were considered include (a) knowledge-based method: FREAD; (b) ab initio methods: DISGRO, NGK, and Rosetta-missing-loop (RML); and (c) DL-based methods: AlphaFold RoseTTAFold and ColabFold, which is compatible with AlphaFold 2.3.1 (users can customize the template database). According to the user guide, NGK requires a start conformation of the loop region, and if there is a missing loop, RML can be used to fill it. In addition to these methods, several antibody structure prediction methods were also examined including DeepAb, RosettaAntibody-G [59], RepertoireBuilder [60], AbodyBuilder [61], AlphaFold, and AlphaFold-Multimer to assess the prediction ability of H3 loop region.

The versions of those methods and options are listed below:

FREAD (version 1.0): The “top_models” parameter was configured to 1 for single-conformation evaluations and 5 for multi-conformation evaluations, thereby enabling a streamlined assessment of both single and multi-conformational structures.

DISGRO (original version): The “pdbout” parameter was adjusted to 1 and 5 for various comparison modes, with the other options remaining at their default settings.

NGK and RML: Both integrated within the Rosetta suite (version 3.13), NGK employs the “out:nstruct” parameter, while RML utilizes “nstruct,” both set to 1 for single and 5 for multi-conformation evaluations. The other parameters for NGK matched the integration test standards, whereas RML mirrored the guidelines from the provided demo.

RoseTTAFold (version 1.1.0): The “run_pyrosetta_ver.sh” was chosen for the implementation of monomer structure prediction. This selection yields 5 results, analogous to the outcomes produced by AlphaFold, with the other options kept at the defaults.

AlphaFold (version 2.1.1): Two key configurations were made: the “db_preset” was set to “full-dbs”—a choice that, although time-intensive, ensures the acquisition of highly accurate structures. The “model_preset” was configured for “monomer” to facilitate monomer structure prediction.

AlphaFold-Multimer (version 2.1.1): This method was also evaluated. By choosing “model_preset” as “multimer” and other configurations as default.

ColabFold (version 1.5.2): ColabFold was implemented locally (LocalColabFold), and it was compatible with AlphaFold 2.3.1. By choosing “templates” and “custom-template-path” to the template database we made which consisted of all nonloop structures of CASP13+14 and CASP15 datasets.

Additionally, structural data from DeepAb (version 2.2), RosettaAntibody-G, RepertoireBuilder, and AbodyBuilder were sourced from the pdb-format results supplied by DeepAb.

### Metrics

For loop modeling tasks, it is essential to consider both accuracy and efficiency. Full-atom RMSD was utilized for evaluating the accuracy of prediction. Because AlphaFold, ColabFold, and RoseTTAFold model the entire protein structures, the predicted structures of proteins are aligned before calculating the RMSD values. Additionally, AlphaFold-Multimer, AlphaFold, DeepAb, RosettaAntibody-G, RepertoireBuilder, AbodyBuilder, and RosettaAntibody predict the complete structures of antibodies. Following the same guidelines as DeepAb, the heavy chains of these antibodies are aligned prior to the RMSD calculation. Moreover, the success rate of the method was introduced as a complementary metric to RMSD, which measures the proportion of cases where the RMSD is less than or equal to 2 Å compared to experimentally determined conformations. The efficiency was simply measured by computing time.

### Computing resource

KarmaLoop was trained on 8 NVIDIA A100-SXM4-80GB and 64-core Intel Xeon Platinum 8358P CPU @ 2.60 GHz. For evaluation, KarmaLoop was evaluated on a Tesla V100S GPU. Traditional methods (FREAD, DISGRO, NGK, and RML) were accomplished in parallel with 48-core Intel Xeon Gold 6240R CPU @ 2.40 GHz. AlphaFold-Multimer, AlphaFold, ColabFold, and RoseTTAFold were executed in parallel on 20 cores of the Intel Xeon Gold 6240R CPU @ 2.40 GHz and a Tesla V100S GPU. DeepAb, RosettaAntibody-G, RepertoireBuilder, AbodyBuilder, and RosettaAntibody predicted antibody conformation was derived from DeepAb provided data.

## Data Availability

The source code and testing datasets are available at https://github.com/karma211225/KarmaLoop. The training data information and results are available at https://zenodo.org/records/10046214.
