# Supplementary material for: Highly Accurate and Efficient Deep Learning Paradigm for Full-Atom Protein Loop Modeling with KarmaLoop
Source: Research (Wash D C). 2024 Jul 25;7:0408. doi: 10.34133/research.0408 (PMC11268956; doi:10.34133/research.0408)
Supplement: Supplementary 1 — Figs. S1 and S2 Tables S1 to S3 [file research.0408.f1.docx]

**Supplementary Materials**


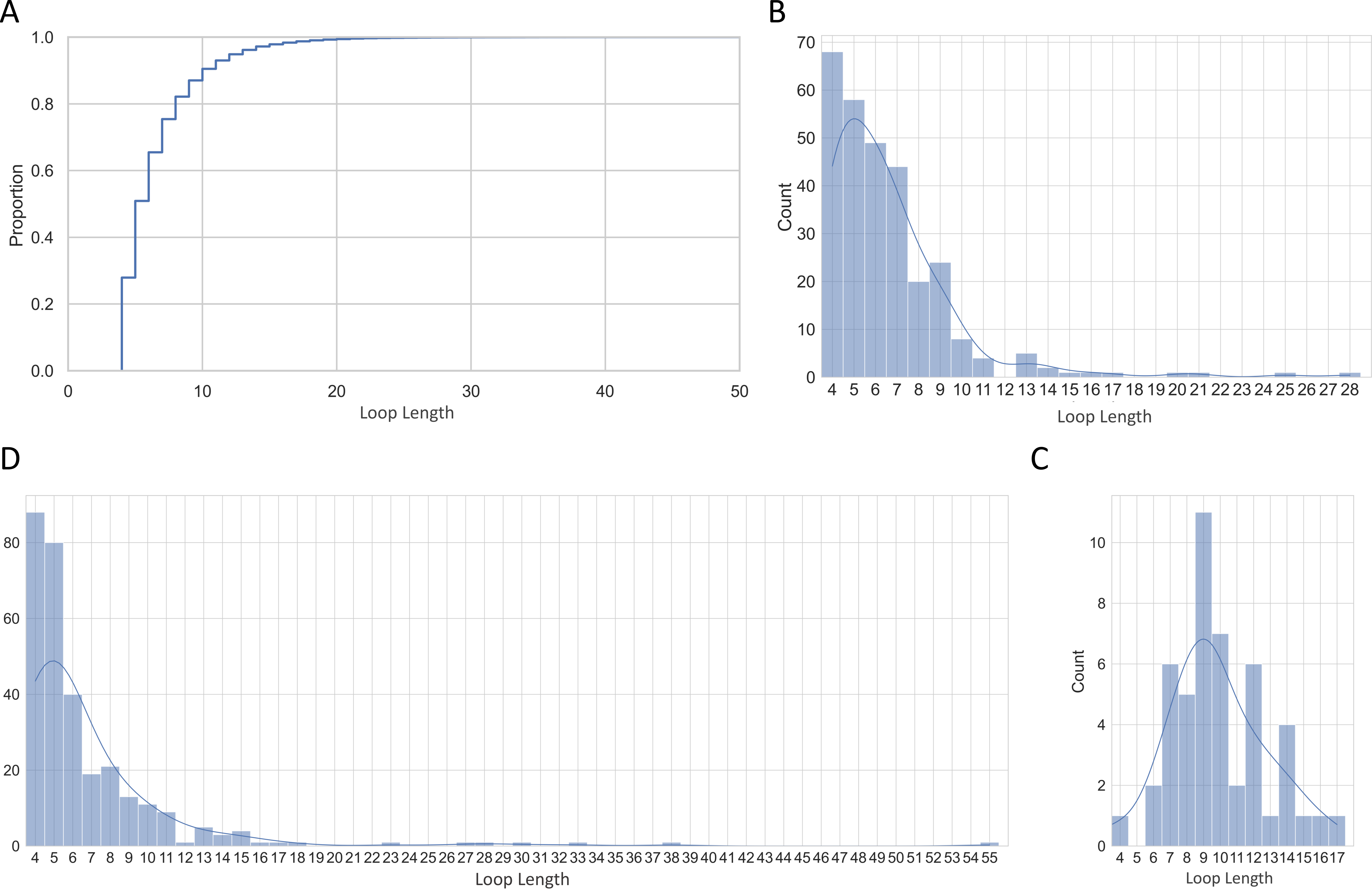


**Figure S1**. Distribution of different loop lengths. (A) Empirical cumulative distribution of the training dataset. (B) Distribution of the CASP13+14 dataset. (C) Distribution of the CASP15 dataset. (D) Distribution of the antibody test dataset.


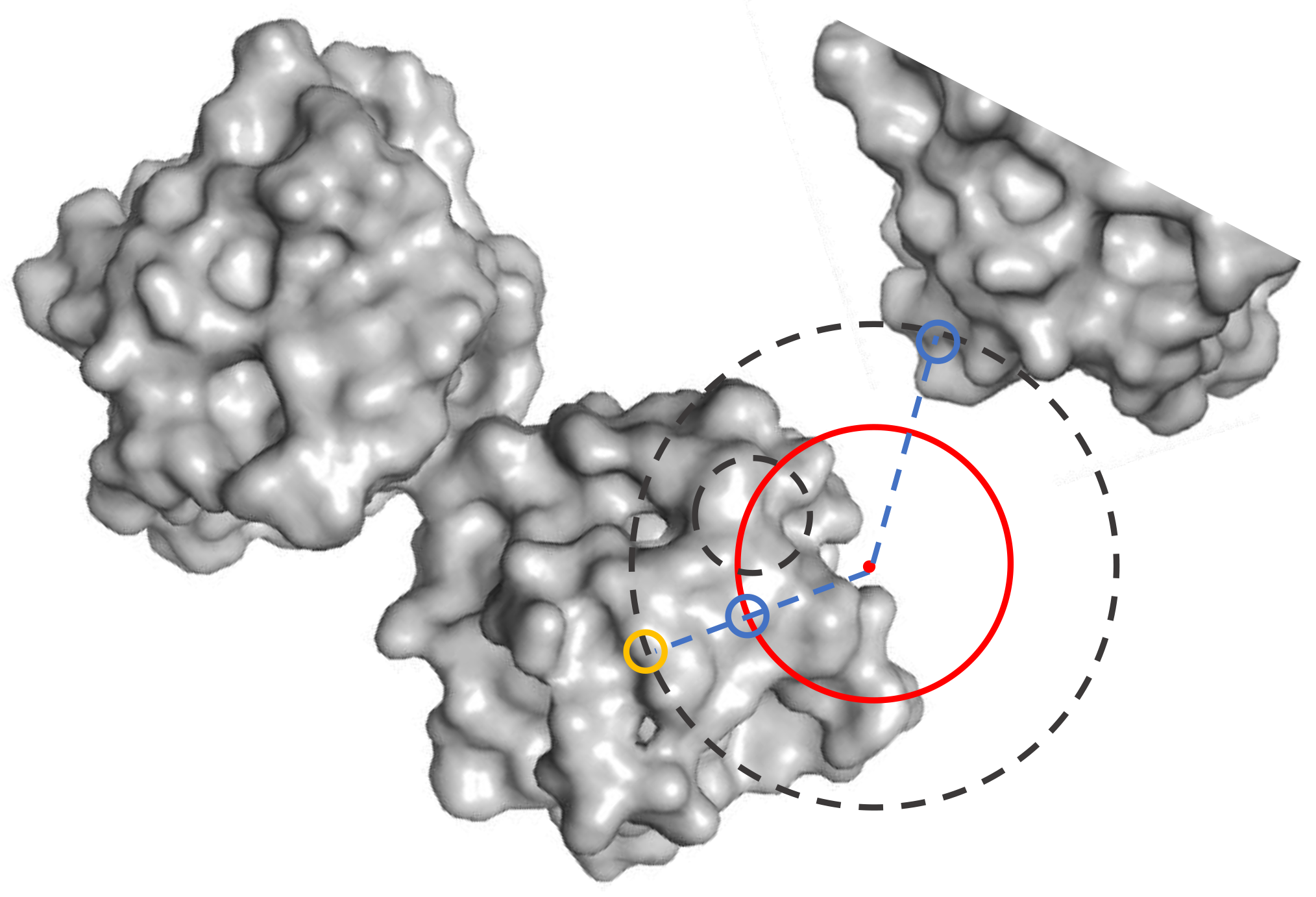


**Figure S2.** Pocket reduction process of KarmaLoop. Red circle indicates *SS (small sphere)*, the bigger black dashed circle indicates *BS (big sphere)*, the smaller black circle indicates the residues located within 5-7 Å of the already selected pocket, blue circle indicates the residue within the *BS*, and the left one was not selected because another residue (yellow circle) in the same direction to the center of the circle was selected. The right blue circle indicates the residue selected as the pocket.

**Supplementary Table 1.** **Node and edge features encoded in compound graphs.**

| Features | Size | Encoding | Description |
| --- | --- | --- | --- |
| Nodes | | | |
| AtomSymbol | 18 | one hot | atom symbol (["H", "B", "C", "N", "O", "F", "Mg", "Si", "P", "S", "Cl", "Cu", "Zn", "Se", "Br", "Sn", "I", "other"]) |
| ChiralTag | 5 | one hot | atomic chiral tag ([0, 1, 2, 3, " other "]) |
| Chirality | 3 | one hot | chirality of an atom ([“R”, “S”, “other”]) |
| TotalDegree | 8 | one hot | the degree of the atom in the molecule including Hs |
| FormalCharge | 12 | one hot | formal charges value in the molecule |
| TotalNumHs | 8 | one hot | the total number of Hs (explicit and implicit) on the atom |
| NumRadicalElectrons | 9 | one hot | the number of radical electrons on the atom |
| Hybridization | 10 | one hot | the atom's hybridization |
| TotalValence | 9 | one hot | the total valence (explicit + implicit) of the atom |
| IsAromatic | 1 | bool2value^a^ | whether the atom is aromatic |
| IsInRing | 1 | bool2value | whether the atom is in a ring with more than 6 atoms |
| IsInRing_3-6 | 4 | one hot | whether the atom is in a ring with atoms range between 3 to 6 |
| Edges | | | |
| BondType | 5 | one hot | the type of the bond (["single", "double", "triple", "aromatic", "other"]) |
| BondDir | 7 | one hot | the direction of the bond |
| Stereo | 7 | one hot | the stereo configuration of the bond |
| IsConjugated | 1 | bool2value | whether the bond is considered to be conjugated |

**Features** denote various atomic and bond properties of the molecules; **Size** indicates the dimension of the feature; **Encoding** demonstrates the method used to represent the feature; **Description** provides detailed information about the features. ***^a^*bool2value** represents the conversion of a boolean feature to a numerical value, where True is represented as 1 and False is represented as 0.

**Supplementary Table 2.** **Node and edge features encoded in protein graphs.**

| Features | Size | Encoding | Description |
| --- | --- | --- | --- |
| Nodes | | | |
| ResidueType | 31 | word  embedding | residue type, (['GLY', 'ALA', 'VAL', 'LEU', 'ILE', 'PRO', 'PHE', 'TYR', 'TRP', 'SER', 'THR', 'CYS', 'MET', 'ASN', 'GLN', 'ASP','GLU', 'LYS', 'ARG', 'HIS', 'MSE', 'CSO', 'PTR', 'TPO', 'KCX', 'CSD', 'SEP', 'MLY', 'PCA', 'LLP', "other"]) |
| ResidueSelfDistance | 5 | float value | distances between atoms inside a residue (maximum and minimum values, between CA and O, between O and N, and between C and N.) |
| ResidueDihedralAngle | 4 | float value | torsion angles values of phi, psi, omega, and chil |
| Orientations | (2, 3) | vector | the forward and reverse unit vectors in the directions of Cα_i+1_ − Cα_i_ and Cα_i−1_ − Cα_i_ |
| SideChains | (1, 3) | vector | the unit vector in the imputed direction of Cβ_i_ − Cα_i_ |
| Edges | | | |
| EdgeType | 1 | bool2value | whether the end nodes of an edge form a covalent bond |
| ResidueDistance | 4 | float value | distance between two residues (between two CA atoms, between two centers of mass, maximum and minimum values) |
| RBFEncoding | 16 | float value | the encoding of residue CA distance with Gaussian radial basis functions |
| SinusoidalEncoding | 81 | float value | a sinusoidal encoding of j – i, representing distance along the backbone |
| EdgeVector | (1, 3) | vector | the unit vector in the direction of Cα_j_ − Cα_i_ |

**Features** denote various residue node and edge properties of the protein; **Size** indicates the dimension of the feature; **Encoding** demonstrates the method used to represent the feature; **Description** provides detailed information about the features.

**Supplementary Table 3.** **Node and edge dim used in KarmaLoop**.

| Features | Size | Description |
| --- | --- | --- |
| Graph representation | | |
| $\boldsymbol{d}_{\boldsymbol{l\_h}}$ | 89 | Loop node features dimension |
| $\boldsymbol{d}_{\boldsymbol{l\_e}}$ | 20 | Loop edge features dimension |
| $\boldsymbol{d}_{\boldsymbol{p\_seq}}$ | 31 | Pocket sequence dimension |
| $\boldsymbol{d}_{\boldsymbol{p\_hs}\boldsymbol{0}}$ | 9 | Pocket nodes scalar features dimension |
| $\boldsymbol{d}_{\boldsymbol{p\_hv}\boldsymbol{0}}$ | 3 | Pocket nodes vector features dimension |
| $\boldsymbol{d}_{\boldsymbol{p\_es}\boldsymbol{0}}$ | 102 | Pocket edges scalar features dimension |
| $\boldsymbol{d}_{\boldsymbol{p\_ev}\boldsymbol{0}}$ | 1 | Pocket edges vector features dimension |
| Graph Transformer (GT) | | |
| $\boldsymbol{d}_{\boldsymbol{h}}$ | 89 | Loop node features dimension |
| $\boldsymbol{d}_{\boldsymbol{e}}$ | 20 | Loop edge features dimension |
| $\boldsymbol{d}$ | 128 | Hidden dimension |
| $\boldsymbol{H}$ | 4 | Number of attention heads |
| $\boldsymbol{d}_{\boldsymbol{k}}$ | 32 | Hidden dimension of each attention head |
| Geometric Vector Perceptrons (GVP) | | |
| $\boldsymbol{d}_{\boldsymbol{seq}}$ | 31 | Pocket sequence dimension |
| $\boldsymbol{d}_{\boldsymbol{hs}\boldsymbol{0}}$ | 9 | Pocket nodes scalar features dimension |
| $\boldsymbol{d}_{\boldsymbol{hv}\boldsymbol{0}}$ | 3 | Pocket nodes vector features dimension |
| $\boldsymbol{d}_{\boldsymbol{es}\boldsymbol{0}}$ | 102 | Pocket edges scalar features dimension |
| $\boldsymbol{d}_{\boldsymbol{ev}\boldsymbol{0}}$ | 1 | Pocket edges vector features dimension |
| $\boldsymbol{d}_{\boldsymbol{hs}\boldsymbol{1}}$ | 128 | Hidden dimension for nodes scalar features |
| $\boldsymbol{d}_{\boldsymbol{hv}\boldsymbol{1}}$ | 16 | Hidden dimension for nodes vector features |
| $\boldsymbol{d}_{\boldsymbol{es}\boldsymbol{1}}$ | 32 | Hidden dimension for edges scalar features |
| $\boldsymbol{d}_{\boldsymbol{ev}\boldsymbol{1}}$ | 1 | Hidden dimension for edges vector features |
| Merge Block | | |
| $\boldsymbol{d}_{\boldsymbol{mv}\boldsymbol{0}}$ | 256 | Merged nodes feature dimension |
| $\boldsymbol{d}_{\boldsymbol{hm}}$ | 256 | Hidden dimension for merged node feature |
| $\boldsymbol{d}_{\boldsymbol{mv}\boldsymbol{1}}$ | 128 | Output nodes feature dimension |
| Interaction graph construction | | |
| $\boldsymbol{d}_{\boldsymbol{h}\mathbf{0}}$ | 128 | Nodes scalar features dimension |
| $\boldsymbol{d}_{\mathbf{e0}}$ | 6 | Edge scalar features dimension |
|  |  |  |
| Mixture Density Network (MDN) | | |
| $\boldsymbol{d}_{\boldsymbol{h}}$ | 128 | Hidden dimension |
| $\boldsymbol{d}_{\boldsymbol{p,c}}$ | 128 | Hidden dimension |
| $\boldsymbol{n}$ | 10 | Number of gaussian distributions |
| E(n) equivariant graph neural network (EGNN) | | |
| $\boldsymbol{d}_{\boldsymbol{h}}$ | 128 | Nodes scalar features dimension |
| $\boldsymbol{d}_{\boldsymbol{e}}$ | 6 | Edge scalar features dimension |
| $\boldsymbol{H}$ | 4 | Number of attention heads |
| $\boldsymbol{d}_{\boldsymbol{k}}$ | 32 | Hidden dimension of each attention head |

**Features** signifies mathematical symbols representing feature dimensions; **Size** indicates the respective dimension values; **Description** provides an explanatory detail about each mathematical symbol's meaning.
